# Host phosphatidic acid phosphatase lipin1 is rate limiting for functional hepatitis C virus replicase complex formation

**DOI:** 10.1371/journal.ppat.1007284

**Published:** 2018-09-18

**Authors:** Lidia Mingorance, Victoria Castro, Ginés Ávila-Pérez, Gema Calvo, María Josefa Rodriguez, José L. Carrascosa, Sofía Pérez-del-Pulgar, Xavier Forns, Pablo Gastaminza

**Affiliations:** 1 Department of Cellular and Molecular Biology, Centro Nacional de Biotecnología-Consejo Superior de Investigaciones Científicas, Madrid (Spain); 2 Department of Macromolecular Structures, Centro Nacional de Biotecnología-Consejo Superior de Investigaciones Científicas, Madrid (Spain); 3 Liver Unit, Hospital Clínic, Institut d'Investigacions Biomèdiques August Pi i Sunyer (IDIBAPS), Consorcio Centro de Investigación Biomédica en Red de Enfermedades Hepáticas y Digestivas (CIBERehd), Universitat de Barcelona, Barcelona (Spain); University of California, San Diego, UNITED STATES

## Abstract

Hepatitis C virus (HCV) infection constitutes a significant health burden worldwide, because it is a major etiologic agent of chronic liver disease, cirrhosis and hepatocellular carcinoma. HCV replication cycle is closely tied to lipid metabolism and infection by this virus causes profound changes in host lipid homeostasis. We focused our attention on a phosphatidate phosphate (PAP) enzyme family (the lipin family), which mediate the conversion of phosphatidate to diacylglycerol in the cytoplasm, playing a key role in triglyceride biosynthesis and in phospholipid homeostasis. Lipins may also translocate to the nucleus to act as transcriptional regulators of genes involved in lipid metabolism. The best-characterized member of this family is lipin1, which cooperates with lipin2 to maintain glycerophospholipid homeostasis in the liver. Lipin1-deficient cell lines were generated by RNAi to study the role of this protein in different steps of HCV replication cycle. Using surrogate models that recapitulate different aspects of HCV infection, we concluded that lipin1 is rate limiting for the generation of functional replicase complexes, in a step downstream primary translation that leads to early HCV RNA replication. Infection studies in lipin1-deficient cells overexpressing wild type or phosphatase-defective lipin1 proteins suggest that lipin1 phosphatase activity is required to support HCV infection. Finally, ultrastructural and biochemical analyses in replication-independent models suggest that lipin1 may facilitate the generation of the membranous compartment that contains functional HCV replicase complexes.

## Author summary

Hepatitis C virus (HCV) infection is an important biomedical problem worldwide because it causes severe liver disease and cancer. Although immunological events are major players in HCV pathogenesis, interference with host cell metabolism contribute to HCV-associated pathologies. HCV utilizes resources of the cellular lipid metabolism to strongly modify subcellular compartments, using them as platforms for replication and infectious particle assembly. In particular, HCV induces the formation of a “membranous web” that hosts the viral machinery dedicated to the production of new copies of the viral genome. This lipid-rich structure provides an optimized platform for viral genome replication and hides new viral genomes from host´s antiviral surveillance. In this study, we have identified a cellular protein, lipin1, involved in the production of a subset of cellular lipids, as a rate-limiting factor for HCV infection. Our results indicate that the enzymatic activity of lipin1 is required to build the membranous compartment dedicated to viral genome replication. Lipin1 is probably contributing to the formation of the viral replication machinery by locally providing certain lipids required for an optimal membranous environment. Based on these results, interfering with lipin1 capacity to modify lipids may therefore constitute a potential strategy to limit HCV infection.

## Introduction

Millions of humans are chronically infected by hepatitis C virus (HCV) worldwide [1]. Chronic HCV infection is a major biomedical problem as it causes liver inflammation and fibrosis, which can lead to severe liver disease, such as cirrhosis and hepatocellular carcinoma [2, 3]. There is no vaccine against HCV and, although blood-screening tests and other prophylactic measures have reduced the dissemination of this pathogen, a number of newly acquired infections still occur associated with risk behavior or with unknown origin [4, 5]. However, chronic HCV infection can be successfully eradicated from chronically infected individuals through specific direct-acting antiviral (DAA) combination therapies, virtually in all treated patients [6]. Since these specific treatments have only been in place recently, there are no sufficient clinical data on the long-term benefit of these treatments in relieving the severity of advanced liver disease [7, 8].

HCV is a Hepacivirus (*Flaviviridae*) with a positive sense, single-strand RNA genome that encodes a single open reading frame (ORF) flanked by untranslated regions (UTR), which are essential for viral polyprotein translation and viral genome replication. HCV ORF is co- and post-translationally processed by cellular and viral proteases to produce ten major proteins. These have been functionally classified in a replication module, that includes the minimal viral components of the RNA replicase (NS3, NS4A, NS4B, NS5A and NS5B) and an assembly module, which comprises the major structural components of enveloped HCV virions, the capsid protein (core) and the envelope glycoprotein complex formed by E1 and E2 heterodimers; as well as the polypeptides p7 and NS2, which are not structural components of virions but contribute to infectious particle assembly in a concerted action with the viral replicase [9].

HCV utilizes key aspects of cellular lipid metabolism for essentially every aspect of the virus replication cycle and strongly interferes with host cell lipid homeostasis [10–12]. In fact, chronic HCV patients display high rates of liver steatosis, severity of which inversely correlates with serum liver derived-lipoprotein [13]. Thus, although host immune response remains a major component in HCV pathogenesis, direct interference of HCV infection with hepatocyte lipid metabolism may contribute to overall disease progression [13].

One of the salient manifestations of HCV interference with lipid metabolism at the cellular level is the formation of a distinctive membranous web in HCV-replicating cells [14]. Within this structure, HCV RNA replication is thought to occur in double-membrane vesicles (DMVs) that emerge from the endoplasmic reticulum [15–17]. HCV replicase formation requires not only viral replicase components NS3 through NS5B [16], but also recruitment and subversion of different key cellular factors that cooperate to provide an optimal membrane microenvironment for the assembly of HCV replicase complexes [11]. In this sense, HCV replicase complexes are located in characteristic detergent-resistant membranes (DRMs) that co-fractionate with caveolin-2 [18–20] and the sigma-1 receptor (SIGMAR1) [21]. These endoplasmic reticulum (ER) microdomains containing the viral replicase are enriched in cholesterol, probably by the direct involvement of cellular proteins involved in non-vesicular cholesterol transport [11].

Assembly of progeny virions is thought to occur at the interphase between ER and lipid droplets (LD) [12] and HCV virion assembly and secretion processes are strongly dependent on host factors involved in biogenesis of triacylglycerol (TAG)-rich lipoproteins [22–25]. Thus, HCV infection requires *de novo* biosynthesis of phospholipids, like phosphatidylcholine (PC), in order to generate its membranous RNA replicase compartments [26] and TAG biosynthesis to ensure progeny virion production [12]. This, together with the fact that PC and TAG biosynthesis are altered by HCV infection [26–28], suggests an important role for glycerophospholipid metabolism in HCV infection.

Lipins are key players in glycerophospholipid metabolism, as they catalyze phosphate removal from phosphatidic acid (PA) to produce diacylglycerol (DAG), which is a precursor of TAG, but also a precursor for PC and phosphatidylethanolamine (PE) biosynthesis in mammalian cells [29]. Lipins may also translocate to the nucleus to directly interact with promoter DNA-binding transcription factors to stimulate or repress transcription of lipogenic genes [30–32]. Two conserved motifs are required for phosphatidic acid phosphatase (PAP) catalytic activity (DXDXT) and transcriptional regulation (LXXIL) respectively [31]. Substrate specificity is restricted to PA, a characteristic that, together with the lack of integral membrane domains and the dependence on Mg^2+^ for catalytic activity, differentiates lipins from other mammalian PAP [33].

Lipin1, the best-characterized member of the family, was discovered as a consequence of the genetic analysis of a mouse strain displaying fatty liver dystrophy phenotype (*fld* mice) [34, 35]. Interestingly, lipin1 deletion in the liver of *fld* mice, results in accumulation of lipin2 protein, a second member of the family predominantly expressed in the liver, which maintains normal cellular PAP activity and compensates some, but not all, aspects of glycerolipid metabolism [36]. Conversely, LPIN2 knock-out mice display increased lipin1 protein expression, as compared with wild type (wt) littermates, suggesting a similar homeostatic compensatory mechanism to maintain liver PAP activity [37]. Thus, lipin1 and lipin2 coordinate lipid homeostasis in the liver [37]. While individual LPIN1 or LPIN2 deletions are tolerated in mice, double knockout mice are embryonically lethal [37]. Despite this apparent functional redundancy, lipin1 is emerging as a key player in ethanol-induced steatosis [38] and a single nucleotide polymorphism in LPIN1 is associated with the severity of liver damage and fibrosis progression in pediatric human patients with histological non-alcoholic fatty liver disease (NAFLD) [39], suggesting that liver lipin1 dysfunction may contribute to steatosis-related liver pathogenesis.

In this study, we focused our attention on lipin1, as analysis of previously published transcriptomic profile datasets revealed that this gene is consistently regulated at the mRNA level during HCV infection. Silencing experiments indicate that HCV infection efficiency is strongly dependent on lipin1 expression. Using different cell culture models of infection, we identified a limiting role of lipin1 PAP activity in the generation of HCV replicase complexes. Defective replicase assembly leads to strong inhibition of HCV propagation in lipin1-deficient cells but not that of another (+) strand RNA virus, indicating a specific role for lipin1 in HCV infection.

## Results

### Lipin1 is rate limiting for HCV infection

**Fig 1:**
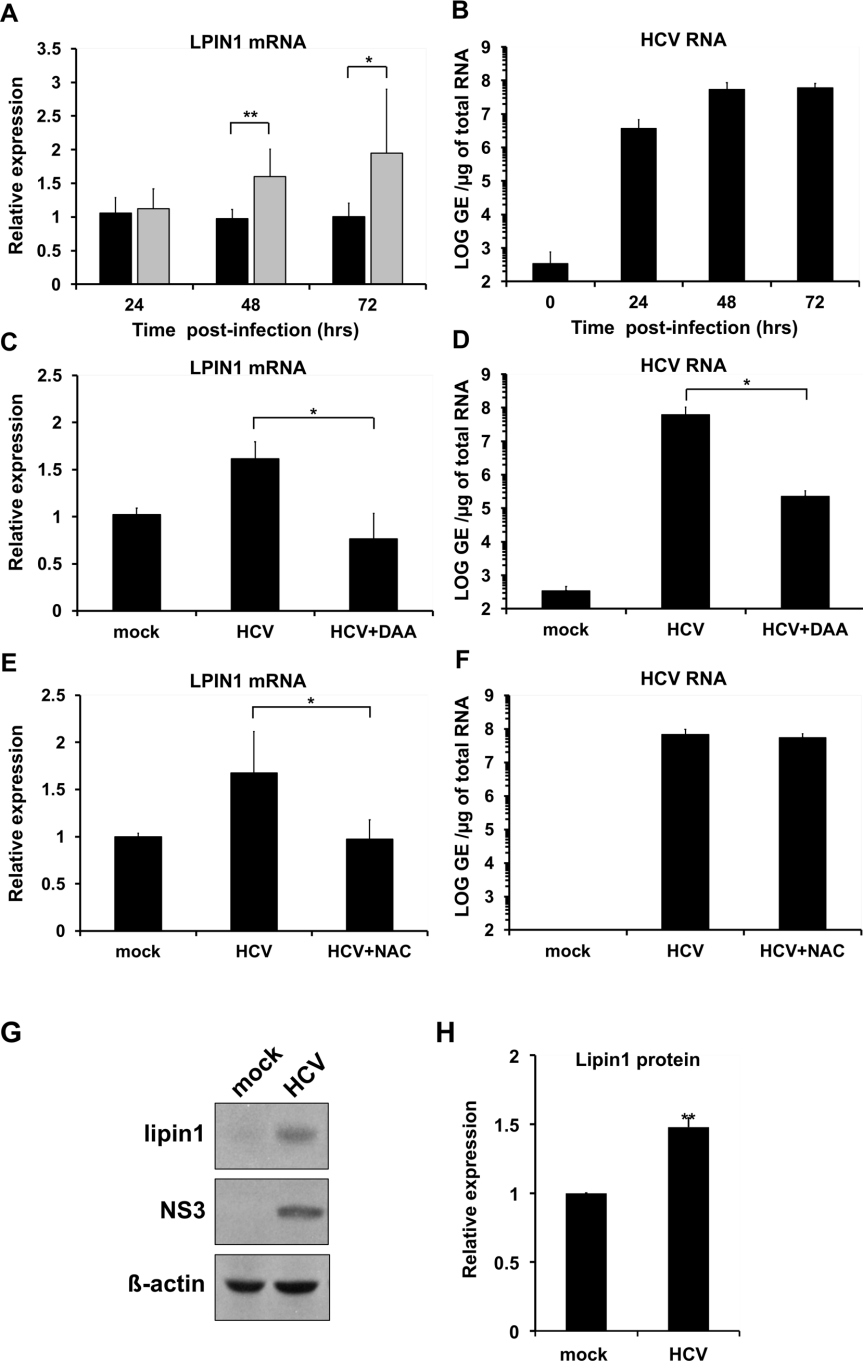
HCV infection induces lipin1 expression. Huh-7 cells were infected at MOI 10 with HCV D183 virus and samples of control (black bars) and infected (gray bars) cells were collected 24, 48 and 72 hours post-infection to determine LPIN1 mRNA levels (A) and intracellular HCV RNA (B). RNAs from mock-infected cells and HCV infected. Impact of sofosbuvir (1μM; DAA) (C, D) or N-acetylcysteine (10mM; NAC) treatment (E, F) on HCV RNA accumulation and LPIN1 mRNA levels. Data are shown as average and standard deviation of two independent infection experiments performed in triplicate (n = 6). G- Protein samples of infected and control cells collected at 48 hours post-infection were subjected to Western-blot analysis to determine lipin1 and NS3 levels, using beta-actin as loading control H-Quantitation of the relative lipin1 protein levels in mock and HCV-infected cells (n = 3). Statistical significance was determined using Student´s t-test (*p<0.05; **p<0.01).

Given the relevance of lipin1 for lipid metabolism and its steatogenic potential, we set out to independently verify data from published differential transcriptomic profile studies that suggested that HCV infection may alter LPIN1 mRNA abundance in cell culture [40, 41]. A specific and statistically significant LPIN1 mRNA induction (Fig 1A) was observed in Huh-7 cells after single cycle infection experiments [multiplicity of infection (MOI) 10] with a cell culture-adapted genotype 2a HCV (D183) variant [42] at the peak of the infection (48 and 72 hours post-infection) and as compared with mock-infected cells (Fig 1B). LPIN1 mRNA induction was prevented when infected cells were treated with 1μM sofosbuvir (Fig 1C), an HCV RNA polymerase inhibitor [43] that reduced viral RNA accumulation by more than two orders of magnitude (Fig 1D), indicating that active HCV replication is required to induce LPIN1 mRNA accumulation.

LPIN1 mRNA has been shown to be upregulated by mechanisms that involve induction of reactive oxygen species (ROS), as treatment of the cells with ROS-scavenger molecule N-acetylcysteine (NAC) is capable of preventing LPIN1 mRNA induction under glucose deprivation [44] or during H_2_O_2_ treatment [45]. Given that transcriptional activation of a subset of lipogenic genes during HCV infection is also prevented by addition of antioxidants [46], we sought to determine if LPIN1 mRNA induction by HCV infection is mediated by ROS production and therefore dampened by NAC treatment. LPIN1 mRNA induction was prevented in the presence of the antioxidant (Fig 1E), despite comparable HCV RNA accumulation in mock-treated and NAC-treated HCV-infected cells (Fig 1F), suggesting that virus replication-induced ROS production is required to induce LPIN1 mRNA accumulation. Western-blot analysis confirmed that the observed transcriptional change leads to a correlative protein accumulation (Fig 1G and 1H); reinforcing the notion that acute HCV infection alters lipin1 expression.

In order to determine if lipin1 subcellular localization was altered during HCV infection, we performed confocal microscopy studies in control and HCV-infected Huh-7 cells. Lipin1 staining was observed as cytoplasmic punctated structures both in control and HCV-infected cells. To study if lipin1 signal colocalized with viral antigens, we performed double staining with antibodies against lipin1 and double-stranded RNA (dsRNA) or replicase subunits NS3 and NS5A. None of the viral antigens strictly colocalized with lipin1 (Pearson´s<0.5) (S1A Fig). However, Mander´s coefficients indicate that the majority of lipin1 overlapped with a small fraction of NS3 and NS5A (S1C Fig). In contrast to lipin1, lipin2 signal did not overlap with that of viral proteins NS3 and NS5A (S1B Fig). Our results indicate that no major lipin1 rearrangements are observed after HCV infection and that only a minor fraction of NS proteins colocalize with lipin1.

**Fig 2:**
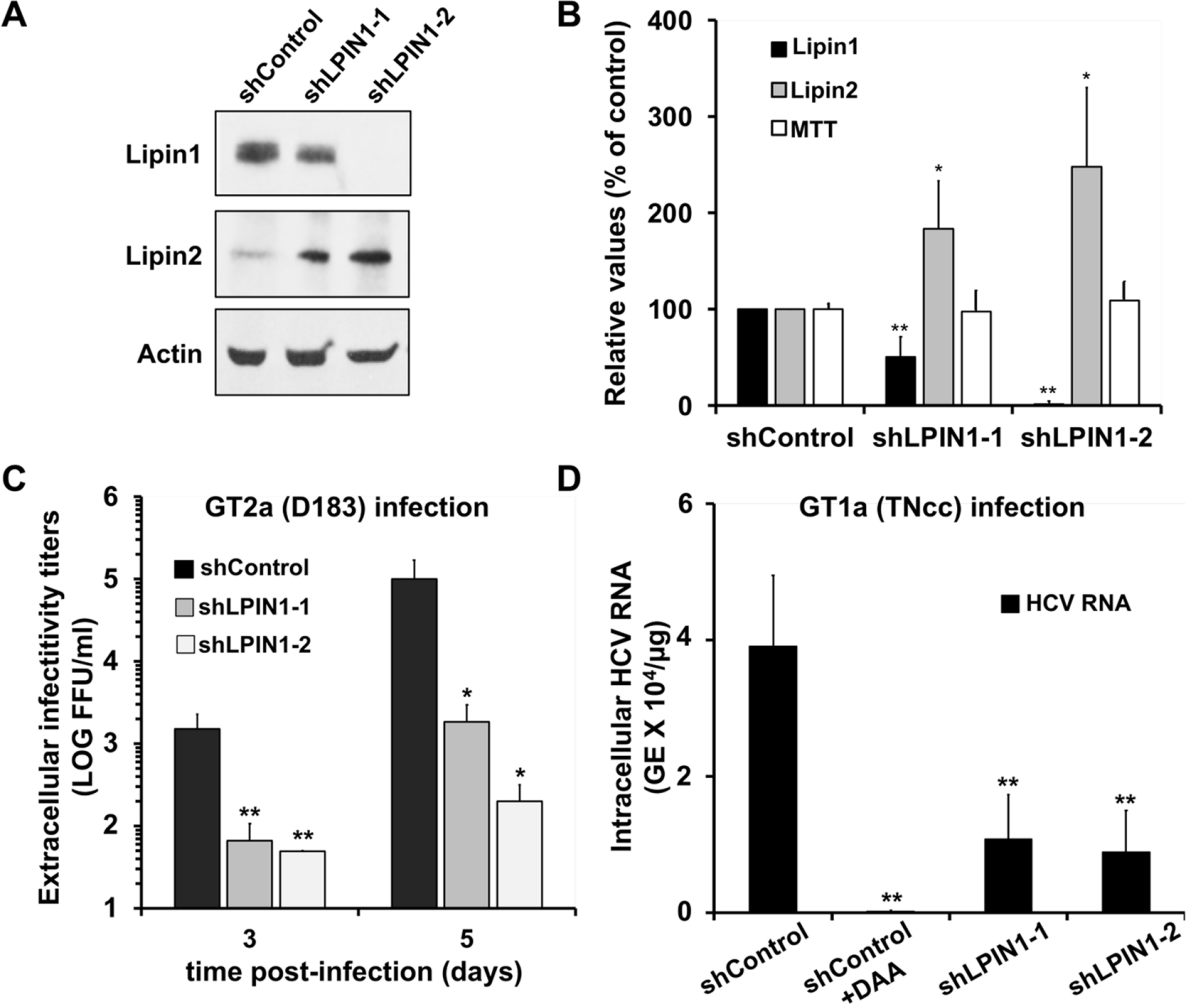
HCV propagation is limited in lipin1-deficient cells. Huh-7 cells were transduced with lentiviral vectors expressing control or LPIN1-specific shRNAs. Seven days after transduction samples of the cells were collected for Western-blot analysis using antibodies against lipin1 and lipin2 and actin as loading control. Parallel cultures were subjected to an MTT assay to determine their viability as described in the methods section. (A) Representative Western-Blot showing cellular lipin1 and lipin2 protein expression levels and a loading control (actin). (B) Average expression values for lipin1 and lipin2 as determined by Western-Blot and cell viability as determined by an MTT assay. Data are shown as average and SD of six independent transduction experiments (n = 6). (C) Huh-7 cells were transduced with lentiviral vectors expressing control or LPIN1-specific shRNAs. At day 3 post-transduction, cells were infected at MOI 0.01 with HCV D183 virus. Samples of cell supernatants were collected at days 3 and 5 post-infection to determine the extracellular infectivity titer. Average and SD of the infectivity titers at day 3 and 5 of two independent infections performed in triplicate (n = 6). (D) Huh-7.5 cells were transduced with lentiviral vectors expressing control or LPIN1-specific shRNAs. Silenced cells were infected 7 days after transduction at MOI 0.05 with genotype 1a HCV (TNcc) in the presence or absence of 2mAde (10μM; shControl+DAA). Intracellular HCV RNA was determined by RT-qPCR 72 hours post-infection. Data are shown as average and SD of three experiments performed in triplicate (n = 9). Statistical significance was determined using Student´s t-test (*p<0.05; **p<0.01).

To study if lipin1 plays any role in HCV infection, lipin1-deficient cells were generated by transducing human hepatoma (Huh-7) cells with lentiviral vectors expressing shRNAs targeting LPIN1 mRNA or a control vector expressing an irrelevant shRNA. Lipin1 expression silencing was verified by Western-Blot, typically 7 days post-transduction (Fig 2A). A partial (50%; shLPIN1-1) and a more profound (>95%; shLPIN1-2) reduction in lipin1 accumulation was observed after transduction with specific shRNAs as compared with the control (Fig 2B). As expected, lipin1 and lipin2 expression is inversely correlated in lipin1 shRNA-expressing cells (Fig 2B). The viability of lipin-deficient cells, as determined by MTT assay [47] was comparable to that of control cells (Fig 2B). These results illustrate that lipin1 shRNA-expressing cells respond homeostatically to functionally compensate partial (shLPIN1-1) and more pronounced (shLPIN1-2) loss of lipin1 (Fig 2B).

Control and lipin1-deficient cells were infected with a genotype 2a D183 at MOI 0.01 to study viral spread by determining extracellular infectivity titers at different times post infection. Fig 2C shows limited propagation of this virus in lipin1-deficient cells, with a statistically significant reduction of up to two (shLPIN1-1) and three orders of magnitude (shLPIN1-2) in viral titer between the control and lipin1-deficient cell lines at day 5 post-infection. These results indicate that lipin1 is required for efficient HCV propagation and that homeostatic lipin2 accumulation (Fig 2B) is not sufficient to support efficient HCV infection.

Since important differences in the interaction of different HCV genotypes with cellular lipid metabolism have previously been described [48], we set out to determine if the observations made with a JFH1-derived virus (genotype 2a) were extensive to other HCV genotypes. We performed low multiplicity infections (MOI 0.05) with genotype 1a TNcc virus strain in lipin1-deficient Huh-7.5 cell lines, which are susceptible to infection by this recombinant virus [49]. First, we verified that Huh-7.5.1 also display a significant reduction in genotype 2a infection efficiency when lipin1 is silenced (S2B Fig). In order to determine TNcc infection efficiency, intracellular HCV RNA accumulation was determined 72 hours post-inoculation in control and lipin1-deficient Huh-7.5 cells. Viral RNA detected under these conditions reflects the ability of genotype 1a to infect and replicate viral RNA in the different cell lines, as treatment of control cells with an HCV polymerase inhibitor 2´-C-methyladenosine (2mAde; 10 μM) [50] reduced viral RNA content by two orders of magnitude (Fig 2D). Lipin1 silencing consistently and significantly reduced TNcc replication by approximately 3-fold both in shLPIN1-1 and shLPIN1-2 cell lines (Fig 2D), indicating that lipin1 is also limiting for genotype 1a HCV infection.

To determine the specificity of these observations, identical lipin1-deficient and control Huh-7 cultures were inoculated at MOI 0.01 with a human alpha-coronavirus CoV-229E bearing a GFP reporter gene (hCoV-229E-GFP) [51]. Inoculation of control and lipin1-deficient cells with this virus resulted in comparable progeny virus production, as determined by infectivity titration in cell supernatants 48 hours post-infection (S3 Fig). These results suggest that lipin1 is not rate limiting for CoV-229E-GFP infection and that lipin1 expression is particularly limiting for HCV.

### Early aspects of HCV infection are limited in lipin1-deficient cells

**Fig 3:**
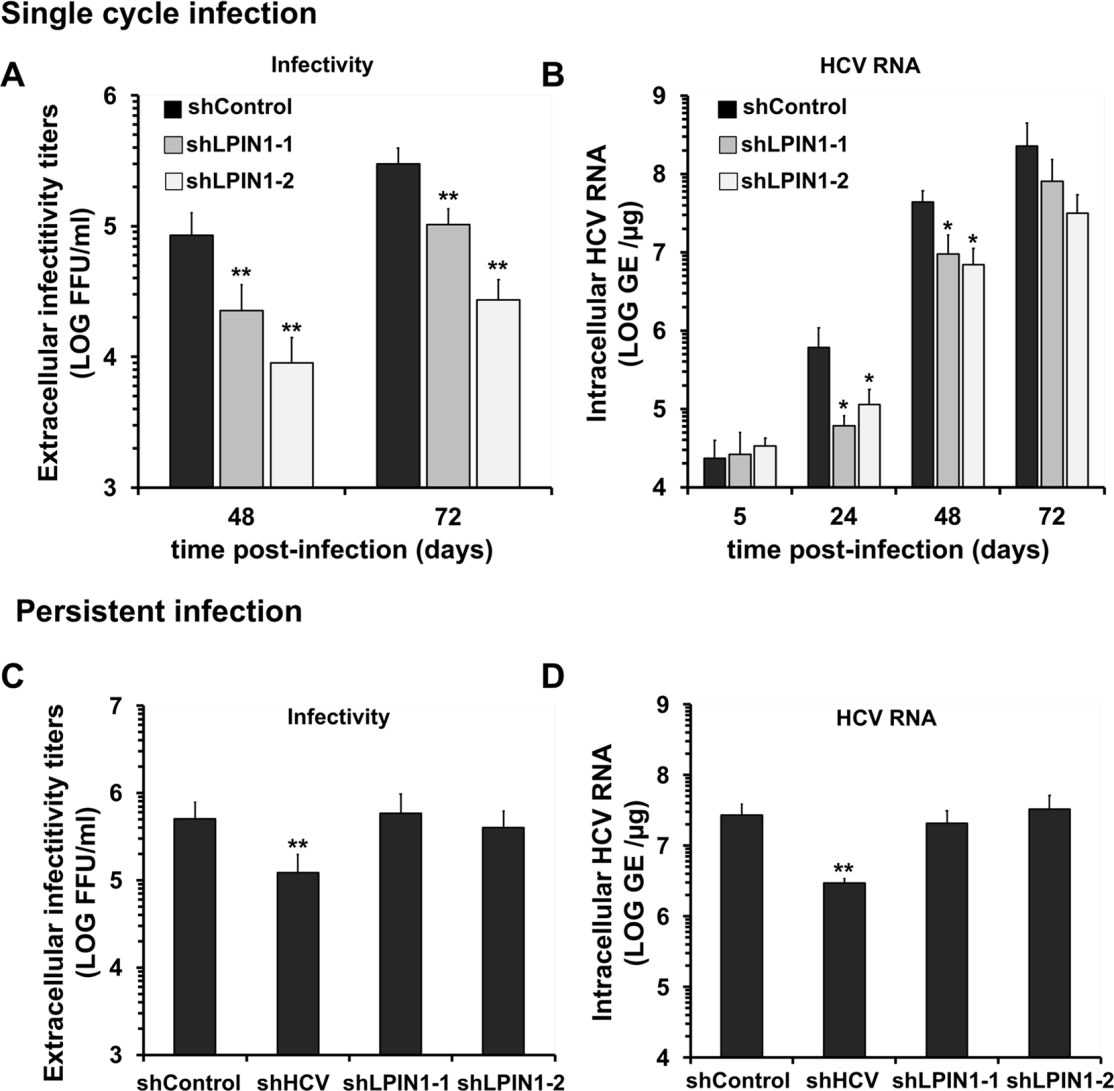
Lipin1 silencing interferes with early steps of HCV infection. Panels A, B- Huh-7 cells were transduced with lentiviral vectors expressing control or LPIN1-specific shRNAs. At day 7 post-transduction, cells were infected at MOI 10 with HCV D183. Samples of the supernatants and the cells were collected at 24, 48 and 72 hours post-infection. (A) Relative infectivity titers found in infected cell supernatants. (B) Intracellular HCV RNA determined at the indicated time points is shown as genome equivalents per microgram of total RNA GE/μg). Panels C, D-Persistently infected cultures were generated by inoculation with JFH-1 virus at MOI 0.01. Once cultures reached >95% of HCV-positive cells, they were transduced with lentiviral vectors expressing control, HCV RNA-targeting or LPIN1-specific shRNAs. At day 7 post-transduction, cells were split and samples of the cells and supernatants were collected 24 hours later to determine infectious virus production rate by infectivity titration HCV (C) and RNA levels by RT-qPCR (D). All data are shown as mean and SD of 3 independent experiments performed in triplicate (n = 9). Statistical significance was determined using Student´s t-test (*p<0.05; **p<0.01).

To determine which aspects of the HCV replication cycle are limited by lipin1 silencing, single cycle infection experiments were conducted by inoculating control and lipin1-deficient cell cultures at MOI 10 with genotype 2a D183 virus. Infection efficiency was measured by titration of progeny virus infectivity present in the supernatant of infected cells and intracellular HCV RNA accumulation at 48 and 72 hours post-infection. Infection of lipin1-deficient cells resulted in a significant reduction of progeny infectious virus production in shLPIN1-1 and shLPIN1-2 cells as compared with the titers observed in the supernatants of control cells (Fig 3A), reinforcing the notion that lipin1 silencing interferes with HCV infection. Reduced virus production is likely due to parallel reduction of intracellular HCV RNA levels observed in lipin1-deficient cells as compared with the control cell line (Fig 3B). This reduction was observed at all time points, except for that at 5 hours, indicating that the size of the inoculum and initial virus adsorption is comparable among the different cell lines (Fig 3B). Thus, lipin1 silencing suppresses HCV infection by interfering with a step of the HCV lifecycle preceding intracellular HCV RNA accumulation.

Next, we set out to determine if lipin1 silencing has any impact on persistent HCV infections to verify if lipin1 is also limiting for late aspects of the virus lifecycle. Persistently infected cells continuously replicate viral RNA, express viral antigens and secrete infectious virions. Thus, it is a valuable system to measure steady-state HCV RNA replication as well as infectious particle assembly and secretion. Persistently infected cultures were transduced with the lentiviral vectors described above to produce persistently infected, lipin1-deficient cells (S4 Fig). Analysis of extracellular infectivity titers revealed that infectivity titers in lipin1-deficient and control cells were comparable, with the exception of a marginal reduction in shLPIN1-2 expressing cells, indicating that lipin1 silencing does not strongly interfere with infectious virus production (Fig 3C). Intracellular HCV RNA levels in lipin1-deficient cells were comparable to that of the control cells (Fig 3D), indicating that lipin1 expression is not rate limiting for HCV RNA replication once infection has been established. Taken together, the results shown above indicate that lipin1 is only limiting at early steps of HCV infection leading to viral RNA accumulation.

**Fig 4:**
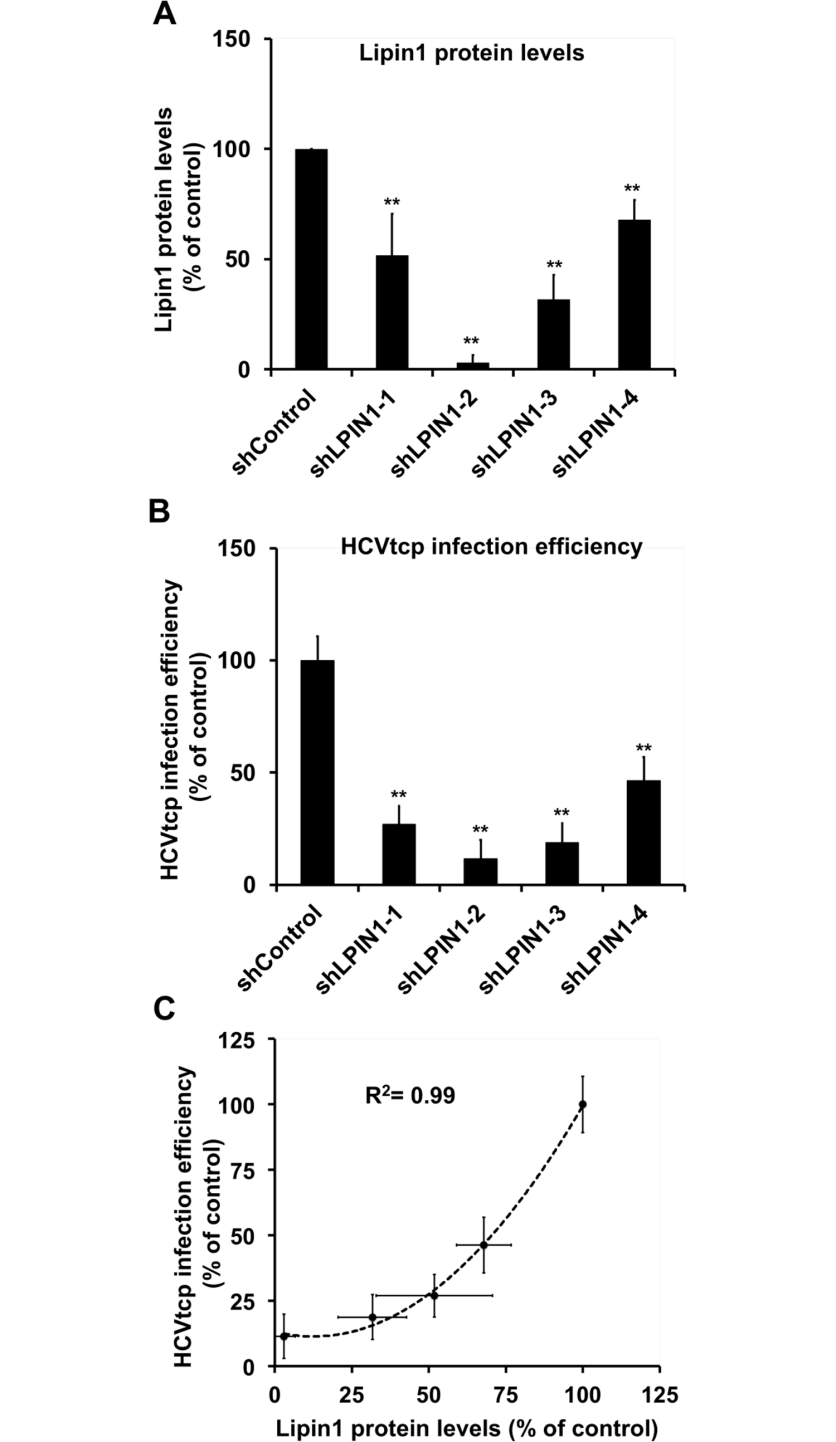
Infection of lipin1-deficient cells with HCV_tcp_. Huh-7 cells were transduced with lentiviral vectors expressing control or LPIN1-specific shRNAs. shLPIN1-1 and shLPIN1-2 target both isoforms, while shLPIN1-3 is specific for lipin1 isoform alpha and shLPIN-4 for isoform beta. Silencing was verified at day 7 post-transduction and control and silenced cells were inoculated with HCV_tcp_. Luciferase activity was determined in the different cell lines 48 hours post-inoculation. (A) Total lipin1 protein quantitation by Western-Blot. (B) Relative HCV_tcp_ infection efficiency. (C) Plot displaying lipin1 expression data versus HCV_tcp_ infection efficiency and the correlation index adjusted to an exponential curve. Data are shown as average and SD of four independent experiments performed in triplicate (n = 12). Statistical significance was determined using Student´s t-test (*p<0.05; **p<0.01).

As an independent verification of the hypothesis that lipin1 is limiting for early aspects of HCV infection, we used a single cycle surrogate infection model based on the production of HCV virions bearing a defective reporter genome encapsidated by trans-complementation (HCV_tcp_). HCV_tcp_ are capable of producing abortive single-cycle infections, efficiency of which is proportional to the luciferase activity found in the target cells [52]. As expected from the results shown in Fig 3, infection of lipin1-deficient cells with HCV_tcp_ resulted in a 75% reduction in the reporter luciferase activity in shLPIN1-1 cells and 90% in shLPIN1-2 determined at 48 hours post-infection, proportionally to the degree of silencing in these cells (Fig 4A and 4B). These results underscore the role that lipin1 plays at early steps of HCV infection and indicate that either viral entry or a step leading to efficient HCV RNA replication is impaired in these cells.

LPIN-1 mRNA is alternatively spliced in human liver to produce isoforms α and β, which may differ in catalytic activity, subcellular localization and gene expression regulation [53]. To determine the relative contribution of these isoforms to HCV infection, isoform-specific shRNAs were generated and used to transduce Huh-7 cells, as described above. LPIN1α-specific shRNA (shLPIN1-3) reduced total lipin1 protein by 70%, while LPIN1β-specific shRNA (shLPIN1-4) reduced total lipin1 expression by only 30% (Fig 4A). Infection of control and lipin1 isoform-deficient cells with HCV_tcp_ revealed that lipin1α silencing resulted in a strong (85%) reduction in HCV infection while lipin1β silencing resulted in a milder (55%) but significant reduction in HCV infection efficiency (Fig 4B). These results indicate that both isoforms alpha and beta are limiting for HCV infection and suggest that the total amount of lipin1 present in the cell determines HCV infection efficiency. Taken together, the results obtained with four different shRNAs indicate that total lipin1 expression levels strongly correlate with HCV_tcp_ infection efficiency (Fig 4C), underscoring a role for this host protein in early aspects of HCV infection.

### Lipin1 silencing interferes with initial steps of HCV RNA replication

**Fig 5:**
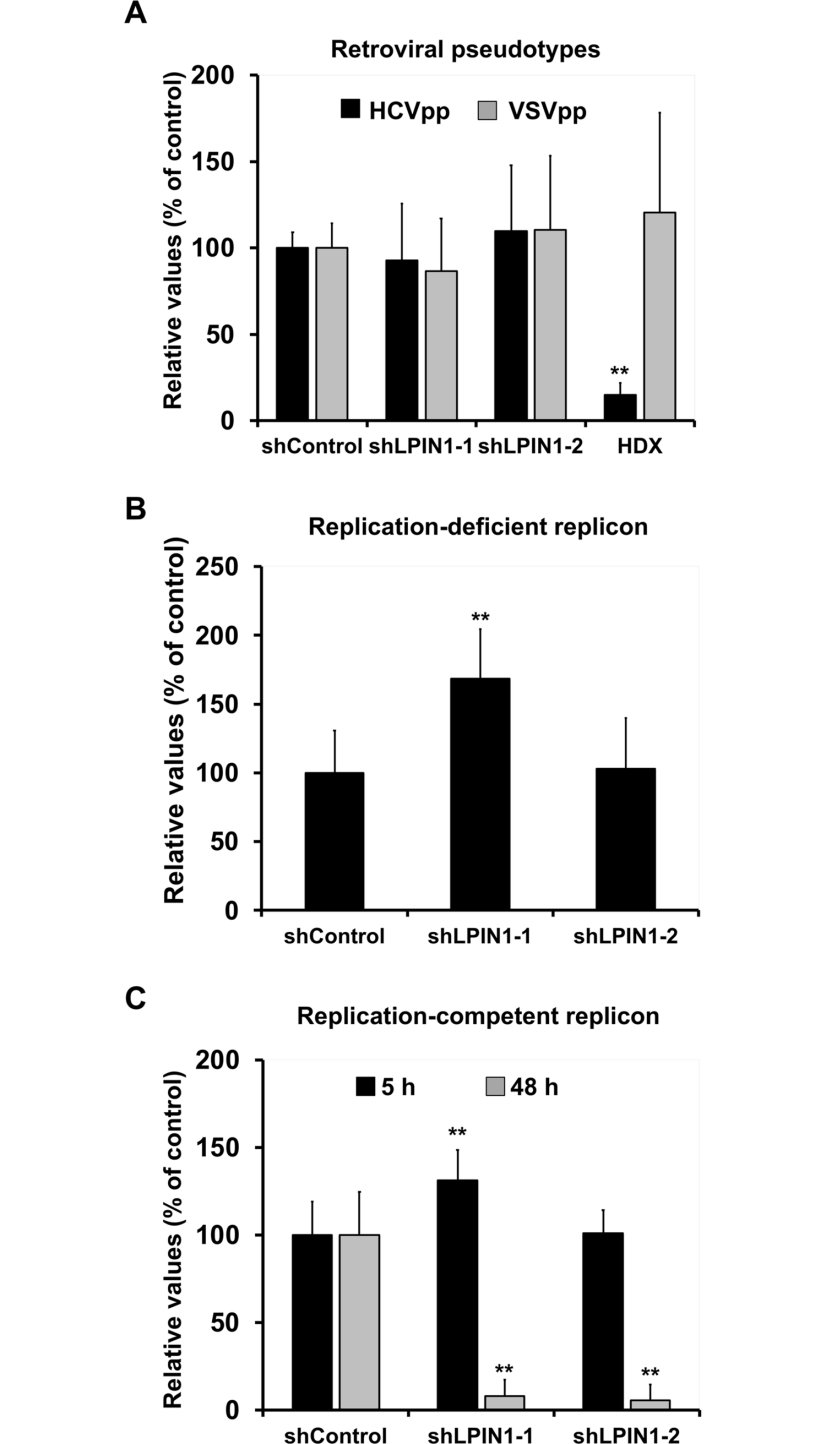
Lipin1-silencing interferes with early HCV RNA replication, downstream of viral entry and primary translation. Huh-7 cells were transduced with lentiviral vectors expressing control or LPIN1-specific shRNAs. (A) Control and silenced cells were inoculated with HCV E1E2 (HCV_pp_) or VSV-G-pseudotyped retroviral vectors (VSV_pp_) 7 days post-transduction. Control cells treated with hydroxyzine (5μM) were used as positive inhibition control. Luciferase activity was determined in the different cell lines at 48 hours post-inoculation. HDX, hydroxyzine pamoate (5μM). Panels B, C- Control and lipin1-deficient cells were transfected with a replication-deficient mutant (B) or replication competent subgenomic HCV replicon bearing a luciferase gene (C). Luciferase activity was determined in the different cell lines at 5 hours post-transfection for both replicons and 48 hours post-transfection for the replication-competent replicon RNA. Data are expressed as average and SD of three independent experiments performed in triplicate (n = 9). Statistical significance was determined using Student´s t-test (*p<0.05; **p<0.01).

Reduced HCV RNA accumulation in a single cycle infection (Fig 3) as well as reduced HCV_tcp_ infection (Fig 4) may be due to a defect in entry of incoming virions. HCV E1E2-pseudotyped retroviral vectors bearing a luciferase gene (HCV_pp_) were used to measure viral entry because they constitute a sound model to study viral adsorption, receptor-mediated internalization and E1E2-mediated fusion in endosomes [54]. To assess the specificity of these observations, parallel cultures were inoculated with VSV-G pseudotyped retroviral vectors (VSV_pp_). Control and lipin1-deficient cell lines were infected with HCV_pp_ (genotype 2a; JFH-1 strain) and VSVpp. As a positive control of inhibition of HCV entry, we used hydroxyzine (5 μM), which efficiently blocks HCV infection by interfering with viral entry [55]. As expected, hydroxyzine selectively inhibited HCV_pp_ infection, as shown by reduced luciferase levels 48 hours post-inoculation only in HCV_pp_-infected cells (Fig 5A). Interestingly, lipin1-deficient cells (shLPIN1-1 and shLPIN1-2) were fully susceptible to HCV_pp_ and VSV_pp_ infection, as comparable luciferase activity levels were found in all cell lines 48 hours post-inoculation (Fig 5A). These results indicate that lipin1 is not rate limiting for receptor binding, particle internalization or E1E2-mediated endosomal fusion, which are steps recapitulated in this model [54]. Based on the data presented thus far, we hypothesized that lipin1 is limiting for a step in the HCV lifecycle downstream of HCV entry, leading to HCV RNA accumulation. To verify the hypothesis that lipin1 silencing causes strong reduction in initial HCV RNA accumulation by interfering with a step downstream of viral entry, we bypassed this step of the viral replication cycle by transfecting a subgenomic HCV RNA replicon bearing a reporter luciferase gene into control and lipin1-deficient cells. First, we evaluated HCV-IRES driven primary translation of incoming genomes by transfection of a replication-deficient mutant replicon that bears an inactivation mutation in the catalytic site of NS5B RNA polymerase [56]. Luciferase activity measured at 5 hours post-transfection was not reduced in any of the cell lines, indicating that transfection efficiency and HCV IRES-dependent primary translation was not significantly affected by lipin1 silencing (Fig 5B). A significant increase in *Renilla* luciferase activity was observed in shLPIN1-1 cells both when transfecting a replicon (Fig 5B) or a plasmid expressing *Renilla* luciferase under a minimal RNA polymerase II promoter (S5A Fig), suggesting that the increase in luciferase activity observed in these cells is not related with HCV. HCV RNA replication was evaluated by measuring accumulation of a reporter luciferase gene at 5 and 48 hours post-transfection of a replication competent HCV subgenomic replicon. Under these experimental conditions luciferase accumulation at 5 hours also represents HCV IRES-driven primary translation of the input RNA, while reporter luciferase activity at 48 hours depends on effective HCV RNA replication. In contrast to primary translation, HCV RNA replication inferred by luciferase activity at 48 hours was strongly reduced in lipin1-deficient cells (90% in shLPIN1-1 and 99% in shLPIN1-2 cells) (Fig 5C), indicating that initiation of HCV replication is dependent on normal lipin1 expression. This reduction was not due to a non-specific defect in luciferase expression, as co-transfection of a plasmid expressing *Renilla* luciferase lead to comparable luciferase accumulation in all cell lines, discarding the possibility that death of transfected cells or other spurious effects are responsible for the reduced luciferase activity accumulation (S5A Fig). Similar experiments were conducted in ATG4B-deficient cells, as this host factor was shown to be limiting for primary HCV translation [57]. Our studies confirmed that, while partial ATG4B silencing (S5B Fig) significantly interfered with primary translation (S5C Fig) and correlatively with HCV RNA replication efficiency, as expected [57], lipin1-silencing only affected HCV RNA replication (S5D Fig). Overall, these data indicate that HCV RNA replication is not initiated efficiently in lipin1-deficient cells and that blockade occurs at a step downstream translation of incoming HCV genomes.

### Lipin1 phosphatidate phosphatase activity is required to support HCV infection

**Fig 6:**
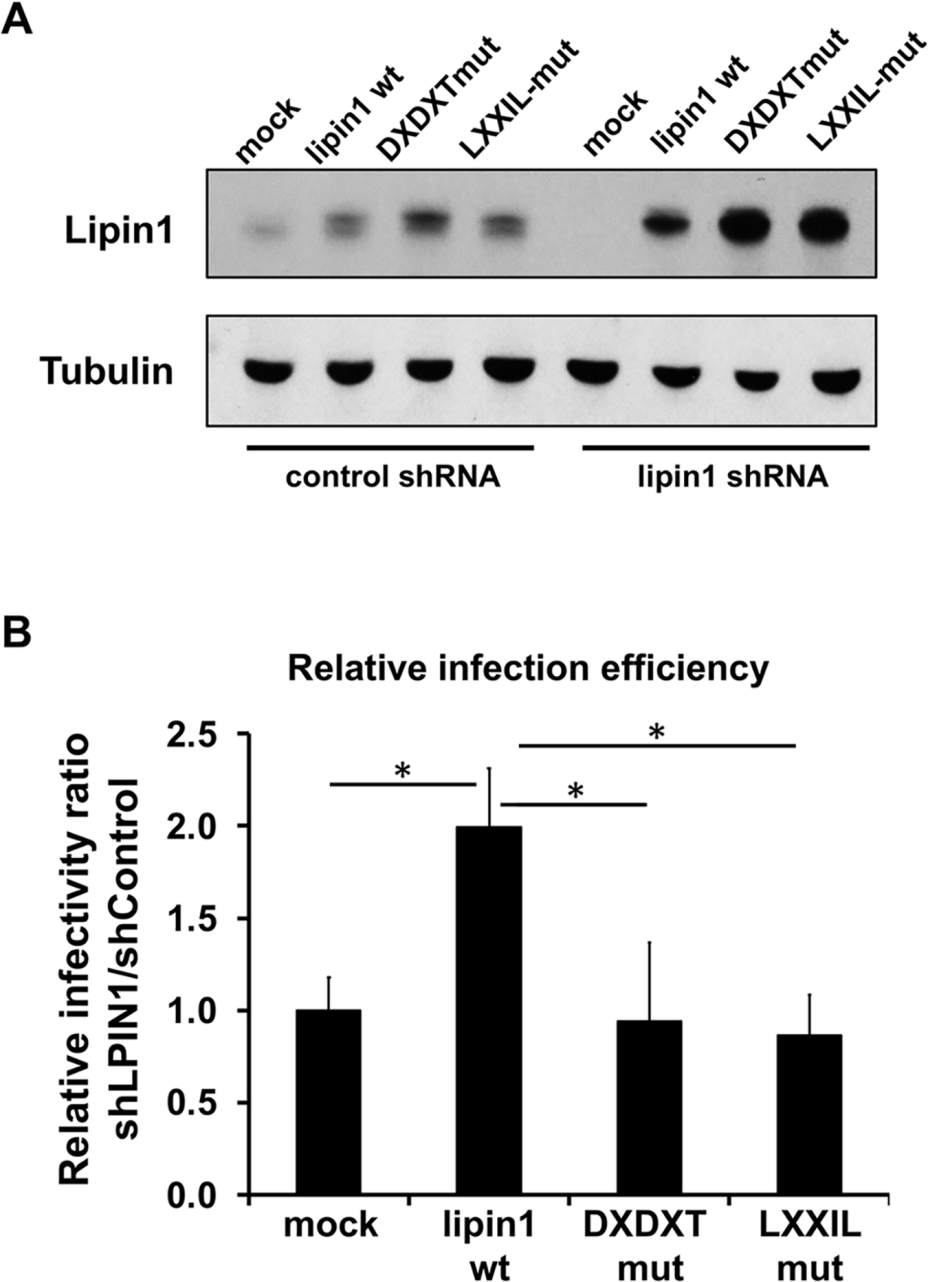
Impact of lipin1 wt and mutant cDNA overexpression in lipin1-deficient cells on HCV infection efficiency. Huh-7 cells were transduced with lentiviral vectors expressing control or LPIN1-specific shRNAs. At day 3 post-transduction, cells were transfected with plasmids encoding wt, DXDXT or LXXIL lipin1beta cDNA. Forty-eight hours later cells were infected at MOI 10 with HCV D183. Extracellular infectivity titers were determined in the supernatants 48 hours post-infection. (A) Western-blot displaying intracellular lipin1 protein levels in the different cell lines at the time of infection, 48 hours post-transfection. (B) Relative infection efficiency in lipin1-deficient cells transfected with lipin1 cDNAs (see main text and S6 Fig for detailed description). Data are shown as average and SD of the infection efficiency relative to mock-transfected cells in two independent experiments performed in triplicate (n = 6). Statistical significance was determined using Student´s t-test (*p<0.05; **p<0.01).

In order to determine if any of the known functions ascribed to lipin1 is required for HCV infection, we tested the ability to restore HCV infection susceptibility of silencing-resistant wild-type (wt) or mutant lipin1 versions bearing a mutation in the catalytic site responsible for its phosphatase activity (DXDXT) and a mutant in the LXXIL motif, which is inactive both for transcriptional activation as well as for phosphatase activity [31]. Control and lipin1-deficient cells were transfected with wt lipin1 beta cDNA as well as with DXDXT and LXXIL mutants. Comparable overexpression levels of the wt and mutant proteins was obtained in each cell line, although overexpressed lipin1 levels were consistently higher in lipin1-deficient cells (Fig 6A). Cells were subsequently inoculated with HCV D183 at MOI 10 and relative infection efficiency was calculated by determining extracellular infectivity titers 48 hours post-infection. Overexpression of wt lipin1 did not significantly alter susceptibility to HCV infection in control cells, although we observed a small but consistent reduction in extracellular infectivity titers when overexpressing wt and mutant lipin1 constructs (S6A Fig). In contrast to control cells, wt lipin1 overexpression in lipin1-deficient cells consistently increased infectivity titers as compared to mock-transfected cells (S6B Fig) and in clear contrast with the reduction observed in control cells (S6A Fig) or when overexpressing mutant lipin1 in lipin1-deficient cells (S6B Fig). In order to take into account this divergent behavior in control and lipin1-deficient cells, we calculated the relative infection efficiency as the ratio of the infectivity titers found in lipin1-deficient cells and control cells (S6C Fig). Ratios were subsequently normalized to that found in mock-transfected cells in order to average the data from different experiments (S6 Fig). Using relative infection efficiency as readout of this set of experiments, we could clearly observe a statistically significant increase (2-fold) in the relative infection susceptibility in cells overexpressing wt lipin1 cDNA as compared with mock-transfected cells or cells expressing similar or higher levels of the mutants (Fig 6A), suggesting that wt lipin1 modestly, though significantly, rescues HCV infection while phosphatase (DXDXT) and transcriptional coactivation (LXXIL) mutants do not (Fig 6B). These results suggest that lipin1 transcriptional co-activation capacity is not sufficient to support HCV infection while lipin1 phosphatase activity is essential. However, given that LXXIL mutant is deficient both in transcriptional co-activation and PAP activity [31], we cannot determine if transcriptional co-activation by lipin1 is also required to support HCV infection.

### HCV replicase complex formation is reduced in lipin1-deficient cells

The data described above suggest a role for lipin1 phosphatase activity in a step of HCV replication cycle between translation of the viral genome and formation of functional replicase complexes. Data regarding primary translation where inferred from a surrogate model of translation based on a reporter luciferase gene (Fig 5B). To address if indeed viral polyprotein is properly processed and inserted into detergent-resistant microdomains to form the characteristic membranous ultrastructures bearing the viral replicase, we used a replication-independent surrogate model of polyprotein expression. This system is based on a vector encoding the portion of the viral polyprotein corresponding to the replicase (NS3-NS5B) under the transcriptional control of the T7 polymerase and the translational control of encephalomyocarditis virus (EMCV) IRES (pTM-NS3/5B) [58]. Replication-independent polyprotein overexpression systems enable assessment of polyprotein processing as well as studying the formation of virus-derived membranous structures [16, 59].

**Fig 7:**
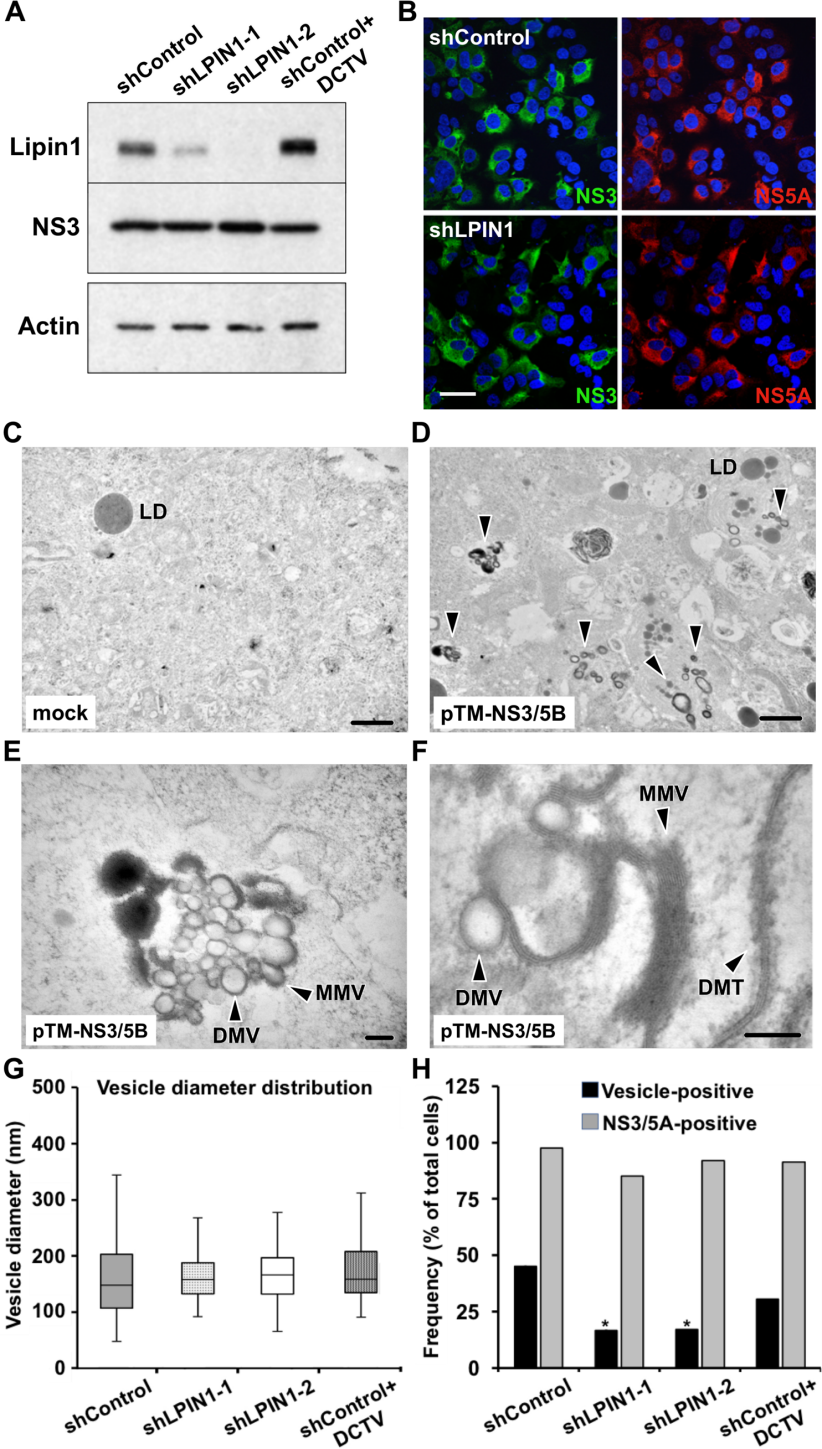
HCV-induced vesicular structure formation is reduced in lipin1-deficient cells. Huh-7 cells were transduced with lentiviral vectors expressing control or LPIN1-specific shRNAs. HCV polyprotein was expressed in the different cells by vaccinia T7 infection and pTM-NS3/5B plasmid transfection at day 7 post-transduction. Samples were collected 16 hours post-infection for Western Blot, immunofluorescence confocal microscopy and TEM. (A) Polyprotein expression efficiency was determined by Western-Blot using an antibody against NS3 and beta actin as loading control. B- Immunofluorescence microscopy showing comparable transfection efficiency and expression of NS3 and NS5A replicase subunits in control and lipin1-deficient cells. Scale bar 50 μm. Panels C, D- Low magnification TEM images from VacT7-infected, mock-transfected (C) and HCV polyprotein-expressing cells (D). LD, lipid droplet. E, F-Higher magnification images of HCV-polyprotein-expressing cells showing DMV, MMV and DMT structures. Scale bars 1μM (panels C, D), 200 nm (panel E), 100 nm (panel F). G- Whisker plot of HCV vesicle diameter distribution in the different cell lines (shControl n = 279; shLPIN1-1 n = 24; shLPIN1-2 n = 61; shControl+DCTV n = 93). Data were collected from three independent experiments. Box plots represent median and quartiles (Q1 and Q3). Whiskers represent 1.5 interquartile range (IQR) above or below the corresponding quartile. (H) Cells were analyzed by immunofluorescence microscopy to determine the percentage of NS3/NS5A-positive cells (shControl n = 84; shLPIN1-1 n = 115; shLPIN1-2 n = 75; shControl+DCTV n = 122). The same samples were analyzed by TEM to determine the presence/absence of vesicular structures (HCV vesicle-positive) in individual to determine the relative incidence of these structures in the different cell lines (shControl n = 31; shLPIN1-1 n = 36; shLPIN1-2 n = 41; shControl+DCTV n = 42 cells). Data are shown as percentage of total cells. DCTV, daclatasvir 100nM. Statistical significance was determined using Fisher´s Exact Test and Chi Square Test (*p<0.05; **p<0.01).

Control and lipin1-deficient cells were infected with a recombinant vaccinia virus expressing T7 RNA polymerase (VacT7) and subsequently transfected with the plasmid pTM-NS3/5B to enable viral replicase expression. Sixteen hours post-transfection, cells were processed for Western-Blot using anti-NS3. Accumulation of NS3 is comparable in control and both lipin1-deficient cells, underscoring the notion that lipin1 is not limiting for polyprotein translation and processing (Fig 7A). Similarly, NS3 and NS5A expression and subcellular distribution was similar in all cell lines (Fig 7B). These results suggest that there are no major differences in accumulation of viral proteins in lipin1-deficient cells and that a step downstream is affected in these cells.

Transmission electron microscopy (TEM) of ultrathin cell sections of cells expressing HCV replicase components shows the expected accumulation of a mixture of characteristic double-membrane vesicles (DMV) as well as multiple membrane vesicles (MMV) (Fig 7D, 7E and 7F) that were not found in mock-transfected cells (Fig 7C), as reported in previous studies using similar systems [16]. Individual vesicle diameter displays heterogeneous size distribution in which the predominant population is distributed between 125–150 nm (median 150 nm; average ± SD: 167 ± 81 nm, n = 279) with larger vesicles being less predominant (Fig 7G and S7 Fig). This size distribution is compatible with that observed similar replication-deficient systems and during HCV infection. Treatment of these cells with 100 nM daclatasvir (DCTV), resulted in a strong reduction in the number of vesicles per section area (S7B Fig), without significantly altering the size distribution of the remaining vesicles (Fig 7G), as reported by Berger et al. [59]. Similarly, the diameter distribution of the vesicles found in lipin1-deficient cells was comparable to that in control cells (Fig 7G). However, HCV-induced structures were significantly less abundant 16 hours post-transfection in lipin1-deficient cells than in controls cells (S7C Fig). This reduced abundance is illustrated by a significant reduction in the fraction of cells displaying vesicular structures in lipin1-deficient cell cultures (Fig 7H) despite comparable transfection efficiency and viral protein expression levels, indicating that lipin1 may be required in a critical step leading to formation of the HCV-induced vesicular compartment.

**Fig 8:**
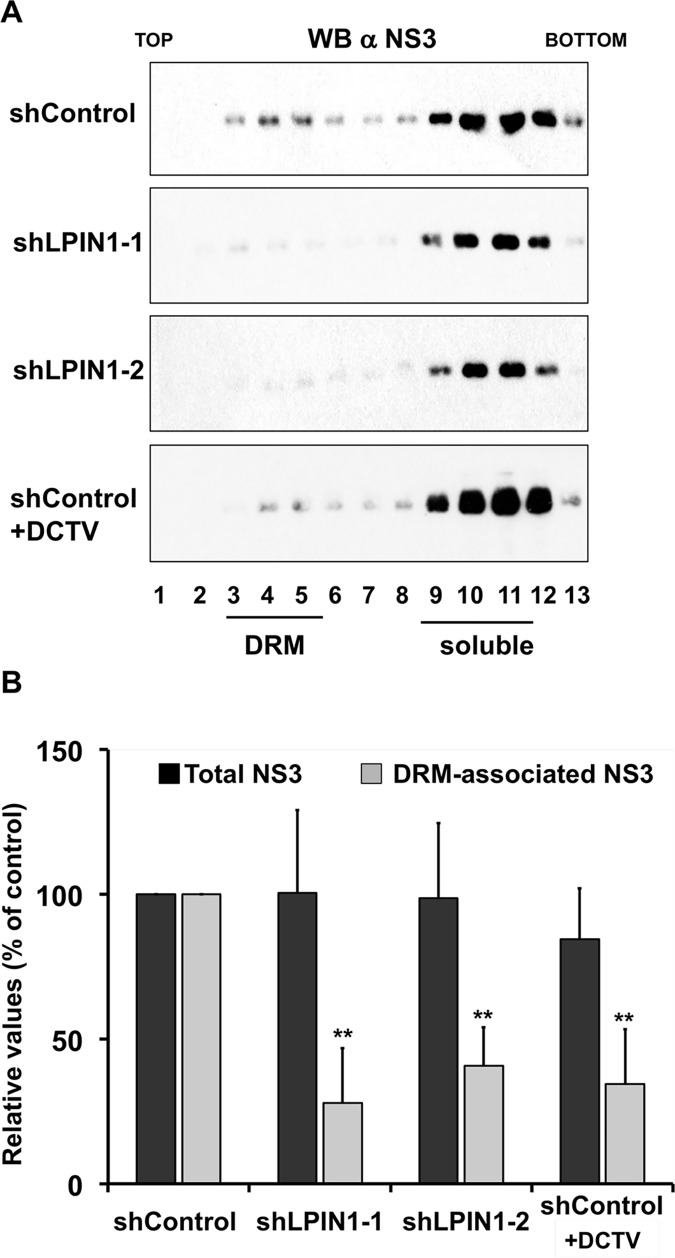
Reduced NS3 incorporation into detergent-resistant membranes (DRM) in lipin1-deficient cells. Huh-7 cells were transduced with lentiviral vectors expressing control or LPIN1-specific shRNAs. HCV polyprotein was expressed in the different cells by vaccinia T7 infection and pTM-NS3/5B plasmid transfection at day 7 post-transduction. Sixteen hours post-transfection, cells were lysed in TNE-0.1% Triton X114 and clear cell lysates were subjected to isopycnic ultracentrifugation in 10–40% sucrose gradients at 120, 000g during 16 hours at 4°C. Gradient fractions were subjected to SDS-PAGE and Western Blot to determine NS3 distribution. (A) Representative Western-Blot of gradient fractions showing the distribution of NS3 in control and lipin1-deficient cells. (B) Quantification of the total and DRM-associated NS3 in the different cell lines, including a control of shControl cells treated with 100nM daclatasvir (shControl+DCTV). Data are expressed as the relative value as compared with the control shRNA. Data are shown as mean and SD of four independent experiments (n = 4). Statistical significance was determined using Student´s t-test (*p<0.05; **p<0.01).

To validate the TEM results independently, we set out to establish a biochemical assay to evaluate replication-independent replicase complex formation. One of the characteristics of the HCV replicase complexes is that they are located in detergent-resistant membranes (DRM) [19, 20]. In this sense, NS proteins are associated with replicase complexes that co-sediment with DRM markers such as caveolin-2 or sigma-1 receptor (SIGMAR1) in low-density fractions in isopycnic gradient ultracentrifugation experiments [19, 21]. Control and lipin1-deficient cells were infected with VacT7 and subsequently transfected with limiting doses of the plasmid pTM-NS3/5B [16]. Parallel samples were treated with DCTV (100 nM). Sixteen hours post-transfection, cell lysates were generated and subjected to equilibrium ultracentrifugation in 10–40% sucrose gradients. Gradient fractions were collected and subjected to Western-Blot analysis to determine the impact of lipin1 silencing on NS3, SIGMAR1 and actin sedimentation profiles. Fig 8A shows how, as previously shown for replicon and JFH1-infected cells, NS3 can be detected in DRM fractions (fractions 3–5), as determined by the presence of SIGMAR1 in those fractions (fractions 3–5; S8A Fig), although in this experimental system, unlike during viral infection [21], most NS3 co-sediments together with solubilized proteins, as shown for actin (fractions 9–12; S8A Fig). DRM-associated NS3 is reduced in DCTV-treated and lipin1-deficient cells, while total NS3 expression remains unchanged (Fig 8A). Four independent experiments were performed in which relative-DRM associated NS3, normalized to that found in solubilized fractions, was calculated for each experimental condition (Fig 8B). Lipin1-deficient cells display a consistent and statistically significant reduction in the DRM-associated, but not total NS3 abundance (Fig 8A and 8B; shLPIN1-1 and shLPIN1-2), similar to that observed in control cells in the presence of DCTV (Figs 8A and 8B; shControl+DCTV), consistent with the TEM data (Figs 7 and S7) and the notion that NS3 in DRM fractions may reflect the abundance of replicase complexes formed in these cells. This reduction is not due to an overall reduction in cellular DRM abundance in lipin1-deficient cells, as lipin1-deficient cells display similar SIGMAR1 distribution pattern as the control cells (S8 Fig). Overall, TEM data and DRM floatation assays strongly suggest that lipin1 is rate limiting for the generation of replicase complexes from fully processed polyprotein subunits.

## Discussion

Hepatitis C virus replication cycle is tightly linked to host cell lipid metabolism and interference with cellular lipid homeostasis contributes to viral pathogenesis [3]. One of the most evident consequences of this interference is the high prevalence of liver steatosis among chronically infected patients [13, 60]. This clinical manifestation of the infection has been linked to, among others, chronic ER stress, mitochondrial dysfunction and metabolite depletion induced by HCV infection, which result in the activation of persistent homeostatic adaptation of the cellular lipid metabolism to permit cell survival, at the cost of pathogenic metabolic alterations [11, 61–64].

Among the different regulatory networks that have been shown to be stimulated during HCV infection, PPARα [61], PGC-1α [65], HIF-1 [66] and SREBP [46, 67] have also been shown to regulate transcription of LPIN1 mRNA [31, 45, 68, 69]. Thus, it is likely that stimulation of one or several of these regulatory networks by HCV infection results in the LPIN1 mRNA transcriptional activation observed in this (Fig 1A) and other studies [40, 41]. Importantly, prevention of LPIN1 mRNA accumulation with NAC (Fig 1E) did not significantly interfere with HCV RNA replication (Fig 1F), suggesting that enhanced LPIN1 mRNA accumulation is not required for efficient HCV infection. We favor the hypothesis that ROS induced by HCV protein accumulation actively participates in LPIN1 induction, as treatment with the antioxidant NAC prevented LPIN1 mRNA accumulation, similar to what has been shown for other SREBP-regulated genes during HCV infection [46]. Accumulation of LPIN1 mRNA during HCV infection results in concomitant protein accumulation (Fig 1G and 1H). However, post-translational mechanisms such as phosphorylation, acetylation or sumoylation regulate lipin1 protein stability, membrane association as well as subcellular localization thus influencing the activity of lipin1 as PA-phosphatase and as transcriptional coactivator [70]. Hence, it is difficult to predict the implications of lipin1 protein accumulation during HCV infection. Thus, future studies on the interference of HCV infection with cellular lipin1 functions will be required to determine its role in HCV-related pathogenesis, particularly in its contribution to steatosis.

The data presented in this study provide evidence that lipin1 is rate limiting for HCV infection at an early step of the infection leading to formation of membranous HCV replicase complexes, downstream of viral polyprotein expression and processing. We provide evidence for reduced accumulation of viral RNA during single cycle infection experiments (Fig 2D), that is reminiscent of a faulty initiation of viral replication, as suggested by reduced replication of a transfected subgenomic replicon in lipin1-deficient cells (Fig 5D). Data obtained in replication-independent polyprotein expression models suggest that generation of the membranous compartment that contains functional replicase complexes is severely limited in lipin1-deficient cells, as suggested by a significant reduction of the fraction of cells where these structures could be visualized by TEM (Figs 7 and S7). This hypothesis is further supported by a significant reduction in DRM-associated NS proteins in lipin1-deficient cells (Fig 8), which may reflect limitations in the association of viral replicase subunits with cholesterol and sphingolipid-rich membranes in lipin1-deficient cells [19, 20, 71].

Four different shRNAs targeting LPIN1 mRNA decrease susceptibility to HCV infection proportionally to their ability to reduce total lipin1 protein accumulation (Fig 4). These results, together with the cDNA rescue experiments (Fig 6), strongly reduce the possibility of observing RNAi-associated off-target phenomena. Interestingly, homeostatic accumulation of lipin2 protein in lipin1-deficient cells (Fig 2A and 2B) is not sufficient to compensate for lipin1 loss to support efficient HCV infection. The notion that, despite being capable of mutually compensating basic liver functions [36, 37], lipin1 and lipin2 play non-redundant functions in the liver has previously been proposed [36, 72, 73]. Lipin1 is tightly regulated at many different levels and its activity accommodates PAP activity in response to different physiological situations such as fasting and insulin signaling [70]. Compelling evidence indicates that, while lipin1 and lipin2 cooperate to maintain liver lipid homeostasis, the two proteins differ in many aspects. For instance, lipin1 is transcriptionally induced by PGC-1α and it is also an inducible amplifier of this transcriptional network [31], whereas lipin2 is not [36]. Lipin1 is sumoylated and sumoylation regulates its nuclear localization and function, whereas lipin2 sumoylation could not be demonstrated, despite the presence of a canonical sumoylation motif in its primary sequence [74]. Lipin1 enzymatic activity is blocked by mammalian target of rapamycin (mTOR)-dependent phosphorylation in response to different metabolic stimuli [30, 75], whereas lipin2 is constitutively active even when phosphorylated [76]. Thus, lipin2 is considered more as a constitutive phosphatidic acid phosphatase with lower specific activity than lipin1 [76]. In addition to these differential regulatory networks, it has been shown that *in vitro* PAP activity of purified lipin1 and lipin2 is differentially influenced by the composition of the substrates (liposomes and lipid-detergent micelles) as well as the pH at which the assay is performed [76]. This differential lipid substrate recognition may be reminiscent of the different preferential association with membranes of different subcellular compartments (S1 Fig)[73, 76]. These differences suggest that, while lipin1 and lipin2 may share some common features, they are not functionally interchangeable, particularly not in the case of HCV infection [70].

Our data support the notion that lipin1 silencing has a strong impact on HCV infection without affecting basic cellular functions (Fig 2B) or significantly interfering with infection by an unrelated virus (S3 Fig). Despite great efforts and different overexpression systems, functional rescue of lipin1 functions by wt lipin1 cDNA overexpression in lipin1-deficient cells only lead to a small but consistent rescue of virus infection efficiency, which was only observed when overexpressing wt lipin1 (Figs 6 and S6). Given the multiple transcriptional, post-transcriptional and post-translational regulation levels existing for lipin1 expression, it is conceivable that only a fraction of the overexpressed lipin1 is fully competent to sustain HCV infection. Moreover, high overexpression levels could only be achieved in lipin1-deficient cells (Fig 6A), underscoring the notion that intracellular lipin1 levels are tightly regulated by the host. Nevertheless, overexpression of a mutant lipin1 lacking phosphatase activity (DXDXT) or a mutant inactive as transcriptional coactivator (LXXIL) were not capable of enhancing HCV infection in lipin1-deficient cells as compared with the wt lipin1 (Fig 6B). These data reveal that lipin1 phosphatase activity is required for lipin1 to support HCV infection and suggest that, while transcriptional co-activation by lipin1 may be important, this function is not sufficient to support HCV replication.

Lipins are important enzymes in the main pathway for *de novo* phospholipid biosynthesis by providing DAG derived from the glycerol-3-phosphate pathway to produce PC and PE through the Kennedy pathway [70]. Production of membranous replicase compartments likely requires *de novo* synthesis of PC and/or PE, which are major components of biological membranes that depend on DAG biosynthesis [29]. In fact, local PC biosynthesis is required for efficient replication of (+) RNA viruses and certain PC species accumulate in HCV-infected cells [26, 28]. Although increased lipin2 accumulation may be sufficient to compensate lipin1 silencing at the whole-cell level [37], it is possible that acute, local demand of *de novo* synthesized phospholipids is required at defined suborganellar compartments during early steps of HCV infection and that lipin1-deficiency shortens or alters the availability of different membrane components, demand that may not be satisfied by lipin2, given the differential regulation[76] and subcellular localization of these two proteins (S2 Fig).

Remarkably, deletion of the yeast lipin homologs *pah1/smp2* (*S*. *cerevisae*) or *ned1* (*S*. *pombe*) gene, results in deregulated proliferation of the ER and nuclear envelope membranes [77, 78], with concomitant enhancement in (+) RNA virus replication [79, 80]. The membranous alterations and elevation of total phospholipid content observed in *pah1-*deficient yeast have been ascribed to transcriptional activation of *pah1*-independent alternative phospholipid biosynthetic programs due to PA accumulation [77, 80]. Our data in mammalian cells are more compatible with a shortage of phospholipid production, which may be at the basis of the reduced abundance viral membranous structure (Figs 7 and 8). This opposite outcomes of infection may derive from the fact that yeast use mainly PA (lipin1 PAP substrate) as a precursor for PC biosynthesis, while mammals mainly use DAG (lipin1 PAP product) as precursor for PC biosynthesis through the Kennedy pathway [77, 80–82]. Moreover, in contrast to yeast and lower eukaryotes, which express only one lipin gene, three different lipin genes coordinate glycerolipid homeostasis in mammals [72]. Thus, interfering with expression of one of the members of the family may not be sufficient to observe the same effects observed when deleting *pah1*, as transcriptional and posttranslational homeostatic compensations are in place in mammals, particularly between lipin1 and lipin2 in liver tissue [36, 37]. In this sense, deletion of either lipin1 or lipin2 in mouse models results in a relatively balanced liver phospholipid content while simultaneous deletion of both lipins is embryonically lethal [37]. Accordingly, only minor alterations of ER membranes [83] and no significant alterations in total PC levels [36] have been reported in lipin1-deficient mouse liver. Given the fact that hCoV-229E is fully capable of replicating in these cells (S3 Fig), it is unlikely that a general disruption of *de novo* phospholipid biosynthesis occurs in lipin1-deficient cells, particularly since hCoV-229E infection also induces profound ER membrane rearrangements required for replication [84], some of which are structurally similar to DMVs observed during HCV infection [85]. Thus, we favor the hypothesis that a subcellular pool of glycerophospholipids is managed by lipin1 in Huh-7 cells and that lipin1 silencing perturbs local levels of PA and DAG, limiting local availability of precursors of structural components of virus-induced membranes.

Alternatively, lipin1 deficiency may alter local amounts of important signaling molecules, in particular, that of its substrate (PA) or its product (DAG). Deregulation of the local PA and DAG pools may cause important alterations for the host cell, as both metabolites are potent chemical messengers that regulate different aspects of cellular homeostasis [86–88]. Regarding PA conversion into DAG by lipins, it has been shown that *pah1* (yeast lipin1 homolog) phosphatase activity is critical for transforming local pools of PA into DAG at the ER membrane to facilitate membrane fusion events mediated by SNARE complexes [89, 90]. Mammalian lipin1 phosphatase activity is also critical for transforming local pools of PA that accumulate at the surface of mitochondria to promote mitochondrial fission [91] or at the surface of endolysosomes to facilitate autophagy [92]. Thus, lipin1 and probably other members of the lipin family modulate different aspects of intracellular membrane signaling. Given that the function of host factors known to be involved in functional HCV replicase biogenesis, like VAPA, VAPB and OSBP [11] are indirectly regulated by local PA/DAG pools [93], it is tempting to propose that lipin1 silencing interferes with the function of one or several of these, or other yet uncharacterized cellular factors. In contrast to what has been reported for other host factors required for HCV replicase complex formation [58], we did not find evidence of lipin1 protein relocalization during HCV infection (S1 Fig). Thus, determining the precise mechanism by which lipin1 regulates HCV replicase formation is challenging, as association of lipin1 with different cell membranes is transient and highly regulated by posttranslational modifications [70]. Moreover, some of the known lipin1 cellular functions may be compensated by other lipins, particularly lipin2 in the liver. Nevertheless, our data clearly indicate that lipin1 participates at early stages of HCV replication and that the aforementioned homeostatic compensations by other lipins in regards to cellular metabolism may constitute an advantage when considering lipin1 as a host target for anti-HCV therapy.

## Materials and methods

### Chemicals

HCV antiviral compounds 2´-c-methyladenosine (2mAde), sofosbuvir and daclatasvir were obtained from Boc Sciences (NY, USA), Selleckchem (Texas, USA) and Medchem Express (New Jersey, USA) respectively and dissolved in DMSO to obtain 10mM stock solutions. N-acetylcysteine (NAC) and puromycin were obtained from Sigma-Aldrich (Missouri, USA), dissolved in water to a final concentration of 0.5 M and 50 mg/ml respectively. Hydroxyzine pamoate was purchased from Sigma-Aldrich (Missouri, USA) and dissolved in DMSO to a final 10 mM concentration.

### Cells and viruses

Human hepatoma Huh-7 and derived subclones Huh-7.5, Huh-7.5.1 (clone 2) have been described [94–96] and were kindly provided by Dr. Chisari (TSRI-La Jolla, CA). HEK-293T cells [97] were kindly provided by Dr. Ortin (CNB-Madrid, Spain). Cell cultures were maintained subconfluent in Dulbecco´s Modified Eagle´s Medium (Gibco) supplemented with 10 mM HEPES (Gibco), 100U/ml Penicillin/Streptomycin (Gibco), 100μM non-essential amino acids (Gibco) and 10% fetal bovine serum (Sigma-Aldrich).

#### HCV viruses

Genotype 2a (JFH-1 strain), D183 adapted virus (D183) and genotype 1a (TNcc) virus have been described elsewhere [42, 49, 56]. TNcc plasmid was kindly provided by Dr. Jens Bukh (Huidovre Hospital; Copenhagen, Denmark). Recombinant viruses were generated by electroporation of an *in vitro* transcribed RNA as described previously [42], using clone 2 cells (JFH-1 and D183) virus and Huh-7.5 cells for TNcc. Supernatants containing the infectious virus were used to inoculate naive Huh-7.5.1 clone 2 cells at low multiplicity of infection (MOI 0.01 FFU/cell) to prepare working virus stocks. For TNcc infections, supernatants of electroporated cells were directly used.

#### Lentivirus production

Lentiviral vectors shRNA expression were produced by co-transfection of plasmids pMDL-RRE, pMD2G and pRSV-Rev (a gift from Didier Trono-Addgene plasmids #12251; #12253; #12259), together with the genomic plasmid (Mission pLKO-puro; Sigma-Aldrich) into HEK-293T cells [21]. Selected shRNA target sequences are: CGAGAGAAAGTGGTTGACATA (shLPIN1-1); (CCTCAGACAGAAATGCAGTTT) shLPIN1-2; CACTCCCAGTCCTTCCGGTTC (shLPIN1-3); CCTGTTCCATCCTTCGGAAAG (shLPIN1-4). Plasmids encoding a ATG4B shRNA (ATG4B800) [57], non-targeting shRNA (shControl) as well as an shRNA targeting HCV IRES (shHCV)were previously described [21]. A lentiviral vector plasmid encoding lipin1-alpha cDNA was purchased from Genecopeia (Cat Nbr: EX-T7124-Lv153). To generate lipin1 beta isoform, exon 7 sequence was inserted using NEB-Q5 mutagenesis kit (NEB). Similarly, silent mutations were introduced in shLPIN1-2 target sequence to obtain wt lipin1beta protein expression from a cDNA resistant to silencing. Mutations in the conserved motifs DXDXT (DIDGT>EIDGT) and LXXIL (LGHIL>LGHFF) were also introduced in lipin1beta cDNA using NEB-Q5 kit, based on previously described mutations [31, 98]. The sequence of the entire LPIN1 cDNAs was determined by Sanger sequencing before use, to assess the introduction of only the desired mutations.

#### Coronavirus 229E-GFP

Recombinant human coronavirus 229E-GFP [51] was kindly provided by Dr. Volker Thiel (University of Bern, Switzerland). Virus was propagated by infection (MOI 0.01) in Huh-7 cells. Supernatants were collected at different time points and titrated by end-point dilution and immunofluorescence microscopy.

### Production of lipin1-deficient cell lines

Lentiviral vectors expressing control and LPIN1-specific shRNAs were used to inoculate Huh-7 cells. Twenty-four hours later, cells were subjected to selection with 2.5μg/ml of puromycin to assess the lowest lentivirus dose capable of conferring puromycin resistance to 100% of the cell population. Selected cell populations were subsequently cultured in the presence of puromycin until LPIN1 silencing was ascertained by Western-Blot using anti-lipin1 antibodies, typically at day 6–7 post lentiviral transduction, time at which all experiments were performed in the absence of puromycin. Before execution of all the experiments shown in this study, lipin1 expression was assessed by Western-Blot. Cell viability was determined by a thiazolyl blue tetrazolium blue (MTT) formazan formation assay [47].

### Single and multiple cycle infections with D183 virus

Control and lipin1-deficient cell lines (5. 10^4^ cells/well) were plated onto 12-well plates and were inoculated with D183 virus at a MOI 10 FFU/cell. Samples of the cells and supernatants were collected 24, 48 and 72 hours post-infection. For multiple cycle infection experiments (MOI 0.01), samples of the supernatants were collected at day 3, 5 and 7 post-inoculation. Cells were split 1:3 in the multiple cycle infection experiments at days 3 and 5 to maintain the cultures subconfluent. Extracellular infectivity titers were determined by endpoint dilution and infection foci counting as previously described [99]. Intracellular HCV RNA was determined by reverse transcription and quantitative PCR (RT-qPCR) as previously described [99].

### Western-Blot

Total protein samples were prepared in Laemmli buffer and separated using polyacrylamide denaturing gel electrophoresis (SDS-PAGE). Proteins were subsequently transferred onto PVDF membranes and incubated with 5% milk (lipins) or 3% BSA in PBS-0.25% Tween20 for one hour at room temperature (RT). Primary antibodies against lipin1 (clone B-12; Santa Cruz), lipin2 (H-160; Santa Cruz), NS3 (clone 2E3; Biofront), beta-actin (ab8226; Abcam) and tubulin (clone AA2; Sigma-Aldrich) were diluted in PBS-0.25% Tween20 and incubated for 1 hour (four hours for lipins) at RT. Membranes were subsequently washed for 20 minutes with PBS-0.25% Tween20 three times. Horseradish peroxidase-conjugated secondary antibodies were incubated for 1 hour at room temperature in 5% milk-PBS-0.25% Tween20 and subsequently washed three times for 20 minutes at room temperature. Protein bands were detected using enhanced chemoluminescence reactions and exposure to photographic films. Specific bands were quantitated using the ImageJ Software [100] on non-saturated, scanned films.

### Immunofluorescence confocal microscopy

Huh-7 cells were grown on glass coverslips and infected at high multiplicity (MOI 10) with D183 virus. Forty-eight hours post infection cells were fixed for 20 minutes at RT with a 4% formaldehyde solution in PBS, washed twice with PBS and incubated with an incubation buffer (3% BSA; 0.3% Triton X100 in PBS) for 1 hour. Antibodies were diluted in incubation buffer: rabbit anti-lipin1 antibody (1:50; Cell Signaling-Leiden, The Netherlands), rabbit anti-lipin2 antibody (1:100; H-160; Santa Cruz), mouse anti-dsRNA (1:200; J2 clone; Scicons), anti-NS3 (1:500; 2E3 clone; Biofront) or anti-NS5A(1:200; 7E2 clone; Biofront). Primary antibodies were incubated with the cells for 1 hour (4 hours for lipin2 experiments) time after which the cells were washed with PBS and subsequently incubated with a 1:500 dilution of a goat anti-mouse conjugated to Alexa 488 or Alexa 594 (Invitrogen-Carlsbad, CA). Nuclei were stained with DAPI (Life Technologies) during the secondary antibody incubation using the manufacturer´s recommendations. Cells were washed with PBS and mounted on glass slides with Prolong (Invitrogen-Carlsbad, CA).

Confocal microscopy was performed with a Leica TCS SP8 laser scanning system (Leica Microsystems). Images of 1024 × 1024 pixels at eight bit gray scale depth were acquired sequentially every 0.13–0.3 μm through a 63x/1.40 N.A. immersion oil lens, employing LAS AF v 2.6.0 software (Leica Microsystems). Colocalization indexes were calculated using Jacop plugin for Image J [101] from a minimum of 10 regions of interest (ROI). Images were processed using ImageJ, were medians of 1 pixel were obtained for the different channels, only for illustration, not for analysis. Color levels, brightness and contrast were manipulated for illustration using technical and biological controls as reference.

### RNA extraction and RT-qPCR

Total RNA extraction was performed using the GTC extraction method [102]. Purified RNA (10–500 ng) was subjected to RT-qPCR using random hexamers and a Reverse Transcription Kit (Applied Biosystems). Quantitative PCR was performed using 2X Reaction Buffer from (Applied Biosystems) and specific oligonucleotides as previously described [99, 103]. Standard curves were prepared by serial dilution of a known copy number of the corresponding amplicon cloned in a plasmid vector.

### Infections with genotype 1a TNcc

Control and lipin1-deficient Huh-7.5 cells were inoculated with TNcc virus (MOI 0.05). Due to the relatively low propagation levels of the TNcc virus in this experimental setup, parallel cultures were infected and treated with 2´-C-methyladenosine (2mAde; 10μM) to determine the levels of non-replicative, background HCV RNA. Cells were incubated for 72 hours at 37°C, time after which samples of the cells were collected to determine intracellular HCV RNA levels by RT-qPCR.

### Persistently infected cell cultures

To establish persistently infected cell cultures Huh-7 cells were inoculated at MOI 0.01 with JFH-1 HCV strain as previously described [24]. Cell cultures were maintained subconfluent for two weeks, time after which infection rates reach nearly 100% of the cells, as assessed by immunofluorescence microscopy. At this point cells were split and transduced with the corresponding lentiviral vectors in order to generate lipin1-deficient cell cultures as well as control cell lines. Once silencing had been verified by Western-blot, typically at day 6–8 post-transduction, cells were split and samples of the cells and supernatants were collected 24 hours later to determine intracellular HCV RNA levels by RT-qPCR and extracellular infectivity titers by end-point dilution and immunofluorescence microscopy.

### Production of trans-complemented infectious HCV virions bearing a reporter gene

Infectious, spread-deficient HCV particles produced by trans-complementation (HCV_tcp_) have previously been described [52]. Briefly, Huh-7.5.1 clone 2 cells expressing core-E1 and E2-NS2 regions from JFH-1 by lentiviral transduction, were electroporated with a JFH-1 subgenomic dicistronic replicon bearing a firefly luciferase gene with reagents kindly provided by Dr. Ralf Bartenschlager (U. of Heidelberg). Supernatants containing HCV_tcp_ were collected 36, 48 and 72 hours post-electroporation, pooled and assayed for viral infectivity. HCV_tcp_ infection efficiency was determined by inoculating naïve Huh-7 cells with the electroporation supernatants and measuring luciferase activity 48 hours post-infection using a commercially available kit (Promega).

### Analysis of viral entry using HCV_pp_

Retroviral particle production pseudotyped with different viral envelopes has previously been described [54, 55] with the materials kindly provided by Dr. F. L. Cosset (INSERM, Lyon). Control and lipin-deficient cell lines were inoculated with HCV_pp_ and VSV_pp_ and incubated for 48 hours, time at which total cell lysates were assayed for luciferase activity using a commercially available kit (Promega). A selective HCV entry inhibitor, hydroxyzine pamoate (HDX) from Sigma-Aldrich (Missouri, USA), was used as positive control of inhibition [55].

### In vitro transcription and HCV RNA transfection

A plasmid containing the sequence corresponding to a subgenomic JFH-1 replicon bearing a firefly luciferase reporter gene was kindly provided by Dr. Ralf Bartenschlager (U. of Heidelberg) [52]. After digestion with the restriction enzyme MluI, the linearized plasmid was transcribed *in vitro* using a commercial kit (Megascript T7; Ambion-Paisley, UK). The resulting products were digested with DNAse and precipitated with LiCl. Pelleted RNA was washed with 75% and 100% ethanol, and resuspended in nuclease-free water. *In vitro* transcribed RNA was transfected into the different cell lines together with a plasmid expressing *Renilla* luciferase under a minimal promoter (pRL-null; Clontech-California, USA) using Lipofectamine 2000 and the manufacturer´s recommendations (Life Technologies- California, USA). Firefly and Renilla luciferase activities were measured in the sample using a commercial kit (Dual Luciferase Assay System; Promega-Wisconsin, USA) at different times post-transfection.

### Lipin1 wt and mutant cDNA overexpression in lipin1-deficient cells

Lipin1-deficient cells were generated by lentiviral transduction of shLPIN1-2 shRNA. At day 3 post-transduction, control and lipin1–deficient cell populations (5 X 10^4^ cells/M24 well) were transfected in suspension using lipofectamine 2000 with plasmids (800 ng/M12 well) expressing wt lipin1beta isoform shLPIN1_2-resistant cDNA or DXDXT or LXXIL motif mutants [31]. Transfected cell cultures were incubated for 48 hours and subsequently inoculated at MOI 10 with D183 virus. Infection efficiency was determined by measuring extracellular infectivity titers 48 hours post-infection. Parallel cultures were used to determine relative wt and mutant protein expression efficiency by Western-Blot. Infectivity titers were measured as described above. The relative impact of cDNA expression was estimated by determining the ratio between the infectivity found in lipin1-deficient cells and the control cells transfected with the same plasmid. In order to average experiments with different raw infection efficiency, all the experiments were referenced to the ratio in the mock-transfected cells.

### Human coronavirus infection

Control and lipin1-deficient Huh-7 cells were inoculated with CoV-229E (MOI 0.01) for 2 hours at 37°C. Cells were washed twice with warm PBS and replenished with DMEM-10%FCS. Extracellular infectivity titers were determined 48 hours post-infection by end-point dilution and fluorescence microscopy in Huh-7 cells.

### T7-driven viral polyprotein overexpression

Huh-7 cells were inoculated at MOI 10 with a recombinant vaccinia virus expressing the T7 phage RNA polymerase (VacT7) [104]. Two hours later, cells were transfected with the plasmid pTM-NS3/5B [16, 58] (kindly provided by Dr. Lohmann; U. of Heidelberg) and Lipofectamine 2000 (ThermoFisher-Massachussets, USA) following the manufacturer´s recommendations in terms of total DNA per well (typically 4μg per 35mm dishes with 7.5 X 10^5^ cells/well) and 50% of the recommended lipofectamine:DNA ratio. Transfected cells were cultured in the presence of the DNA replication inhibitor cytosine β-D-arabinofuranoside (AraC; Sigma-Aldrich) for 16 hours to prevent VacT7 replication [105]. When indicated, media was also supplemented with 100nM daclatasvir (DCTV). Total cell extracts were used to determine viral protein accumulation by Western-blot using anti-NS3 antibody (clone 2E3; Biofront) and β-actin (Abcam; ab8226) as loading control.

### Transmission electron microscopy

For ultrastructural electron microscopy studies, control and lipin1-deficient cells expressing NS3-5B polyprotein (see above) were cultured on glass coverslips and fixed *in situ* after polyprotein expression with a mixture of 2% paraformaldehyde (TAAB) and 2.5% glutaraldehyde (TAAB) (1h at room temperature), post-fixed with 1% osmium tetroxide in PBS (45 min), treated with 1% aqueous uranyl acetate (45 min), dehydrated with increasing quantities of ethanol and embedded in epoxy resin 812 (TAAB). Ultrathin, 70-nm-thick sections were cut in parallel to the monolayer, transferred to formvar-coated EM buttonhole grids and stained with aqueous uranyl acetate (10 min) and lead citrate (3 min). Sections were visualized on a Jeol JEM 1200 EXII electron microscope (operating at 100 kV).

Quantitation of HCV-induced structures was performed as follows. To quantitate the differences in total vesicle abundance, TEM sections were visually inspected under the microscope for the presence/absence of vesicular structures. The number of positive cells and total number of cells were inserted in a 2 X 2 contingency table to determine the statistical significance of the differences between control and lipin1-deficient cells using two-tailed Fisher´s Exact Test or two-tailed Chi Square Test. In addition, we determined the frequency of HCV-induced vesicles in DCTV-treated control cells by dividing the number of structures per inspected area and calculating the average and SD of the frequencies found in the different images. The diameters of individual vesicles were determined manually using size-calibrated images and Image J software.

### Detergent-resistant membrane (DRM) ultracentrifugation

Lipin1-deficient and control cells (7.5 X 10^5^ cells) were infected with VacT7 virus (MOI 10) and transfected with limiting doses of pTM-NS3/5B plasmid (typically 800 ng/ well), as higher plasmid doses may difficult observing the reported differences. Cells were lysed by adding 250 μl of TNE (50mM Tris-HCl pH 7.5, NaCl 150 mM and EDTA 2 mM) buffer containing 0.5% Triton X-114 and protease inhibitors (Complete; Roche- Basel, SW). Lysates were incubated for 30 minutes on ice before clearing them by 10-minute centrifugation at 12,000 r.p.m. Clear supernatants were mixed 1:1 with 60% sucrose TNE solution. This mixture was applied on top of a 40% sucrose-TNE cushion and was overlaid with 20% and 10% sucrose-TNE until completing the discontinuous gradient. Gradients were centrifuged for 16 hours at 120,000 X g. Fourteen fractions were collected from the top and analyzed by SDS-PAGE and Western-Blot using antibodies against NS3 (clone 2E3; Biofront), SIGMAR1 (S-18; Santa-Cruz), caveolin-2 (Epitomics; 3643–1) and beta actin as described previously [21]. NS3 signal was quantitated using ImageJ software and the fraction of DRM-associated NS3 was determined as the ratio of NS3 signal in fractions 3, 4 and 5 to the total NS3 signal in the gradient.

## Supporting information

S1 Fig Confocal microscopy does not reveal any major alteration of lipin1-subcellular localization during HCV infection. Huh-7 cells were infected at MOI 10 with HCV D183 virus and samples of infected and control cells were processed for immunofluorescence microscopy using antibodies against lipin1, dsRNA and viral proteins NS3 or NS5A 48 hours post-infection. (A) Representative images of mock and HCV-infected cells showing lipin1 (red) and HCV antigen (green) staining. Nuclei were stained using DAPI. Scale bar 10 μm. (B) Representative images showing lack of colocalization of lipin2 signal with that of viral NS proteins. (C) Colocalization analysis of viral antigens with lipin1 and lipin2 showing Pearson´s correlation as well as Mander´s overlap coefficients. Data are shown as average and SD of analysis of 20 regions of interest (ROI) for lipin1 and 10 ROI for lipin2.
(TIF)

S2 Fig Lipin1-deficient Huh-7.5 cells are less susceptible to HCV infection. Huh-7.5 cells were transduced with control and lipin1-specific shRNA expressing lentiviral vectors. (A) Total protein samples were collected at day 7 post-transduction, serially diluted and subjected to SDS-PAGE and Western-Blot using antibodies against lipin1 and actin as loading control. (B) Lipin1-deficient Huh-7.5 were subjected to genotype 2a HCVtcp infection. Parallel shControl cell cultures were treated with 10μM 2mAde at the time of infection and cultured in the presence of the inhibitor until the end of the experiment (shControl+DAA). Relative infection efficiency is shown as mean and SD of six experiments performed in triplicate (n = 18). Statistical significance was determined using Student´s t-test (*p<0.05; **p<0.01).
(TIF)

S3 Fig Lipin1 silencing does not interfere with human coronavirus virus propagation. Control and lipin1-deficient Huh-7 cells were inoculated with CoV-229E at MOI 0.01. Supernatants were collected 48 hours post-infection and viral spread was estimated by extracellular infectivity titration. Data are shown as average and SD of three independent experiments performed in triplicate (n = 9). Statistical significance was determined using Student´s t-test (*p<0.05; **p<0.01).
(TIF)

S4 Fig Lipin1-silencing is effective in persistently infected cells. Persistently infected cultures were generated by inoculation with JFH-1 virus at MOI 0.01. Once cultures reached >95% of HCV-positive cells, they were transduced with lentiviral vectors expressing control, HCV RNA-targeting or LPIN1-specific shRNAs. At day 7 post-transduction, cells were harvested to verify lipin1 silencing by Western-Blot using antibodies against lipin1 and actin as loading control. Extracts were serially diluted to facilitate quantitation. (A) Representative Western-Blot. (B) Quantitation of lipin1 levels in the different cell lines. Data are shown as mean and SD two independent experiments (n = 2).
(TIF)

S5 Fig Technical and biological controls of replicon transfection experiments. Lipin1-deficient cells were co-transfected with HCV subgenomic replicon bearing *Firefly* luciferase gene and a plasmid encoding *Renilla* luciferase. Dual luciferase activity was measured in samples of the transfected cell lines 48 hours post-transfection. (A) Relative plasmid-derived *Renilla* luciferase as well as SGR replicon-derived *Firefly* luciferase values are shown as mean and SD of two independent experiments performed in triplicate (n = 6). (B) Lipin1 and ATG4B-deficient cell populations (shLPIN1-2 and shATG4B) were produced by lentiviral transduction. Specific silencing was verified by Western-blot in the different cell lines at day 7 post-transduction. Lipin1 and ATG4B-deficient cells were transfected with a replication-deficient mutant (C) or replication competent subgenomic HCV replicon bearing a luciferase gene (D). Luciferase activity was determined in the different cell lines at 5 hours post-transfection for both replicons and 48 hours post-transfection for the replication-competent replicon RNA. Data are expressed as average and SD of three independent experiments performed in triplicate (n = 9). Statistical significance was determined using Student´s t-test (*p<0.05; **p<0.01).
(TIF)

S6 Fig Lipin1 cDNA overexpression in lipin1-deficient cells. Huh-7 cells were transduced with lentiviral vectors expressing control or LPIN1-specific shRNAs. At day 3 post-transduction, cells were transfected with plasmids expressing wt, DXDXT or LXXIL lipin1beta cDNA. Forty-eight hours later cells were infected at MOI 10 with HCV D183. Two independent experiments are shown (left column; Experiment 1 and right column; Experiment 2). Extracellular infectivity titers were determined in the supernatants 48 hours post-infection. Extracellular infectivity titers determined 48 hours post-infection in shControl (A) and shLPIN1 cells (B). (C) Ratio between the infectivity found in shLPIN1 versus shControl cells in each cell line.
(TIF)

S7 Fig Impact of DCTV in the formation of HCV-derived vesicles observed by TEM. **(**A) Vesicle size range distribution in shControl mock-treated cells. (B) Frequency of HCV-related structures in mock or DCTV-treated shControl cells expressed as the number of vesicles per cell section surface (μm^2^). Data are shown as average and SD of 6 (mock-treated) and 10 different cells (DCTV-treated). Statistical significance was determined using Student´s t-test (*p<0.05; **p<0.01). (C) TEM images showing general views of the different cell lines expressing HCV polyprotein (pTM-NS3/5B). DCTV, daclatasvir.
(TIF)

S8 Fig Separation of detergent-resistant membranes from detergent-soluble proteins by isopycnic ultracentrifugation. HCV polyprotein was expressed in shControl cells by VacT7 infection and pTM-NS3/5B plasmid transfection. Sixteen hours post-transfection, cells were lysed in TNE-0.1% Triton X114 and clear cell lysates were subjected to isopycnic ultracentrifugation in 10–40% sucrose gradients at 120, 000g during 16 hours at 4°C. (A) Gradient fractions were subjected to SDS-PAGE and Western Blot to determine distribution of NS3, the detergent-soluble protein beta-actin and the DRM markers SIGMAR1 and caveolin-2 (Cav-2). (B) Isopycnic ultracentrifugation of lipin1-deficient cell lysates showing normal sedimentation of DRM (SIGMAR1) and soluble protein markers.
(TIF)
