# Supplementary figures and images for: Host phosphatidic acid phosphatase lipin1 is rate limiting for functional hepatitis C virus replicase complex formation
Source: PLoS Pathog. 2018 Sep 18;14(9):e1007284. doi: 10.1371/journal.ppat.1007284 (PMC6161900; doi:10.1371/journal.ppat.1007284)

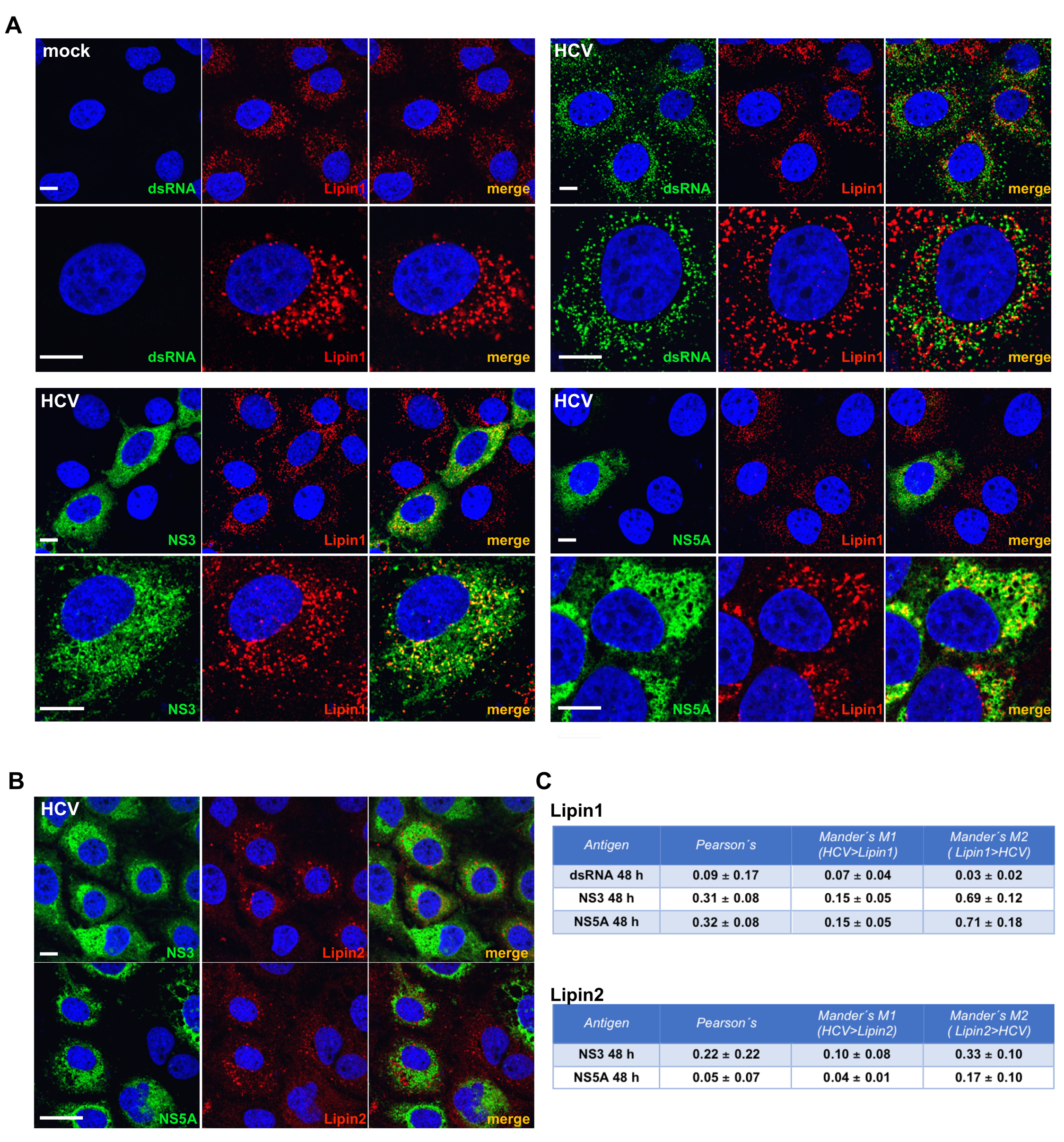

Supplement: S1 Fig — Huh-7 cells were infected at MOI 10 with HCV D183 virus and samples of infected and control cells were processed for immunofluorescence microscopy using antibodies against lipin1, dsRNA and viral proteins NS3 or NS5A 48 hours post-infection. (A) Representative images of mock and HCV-infected cells showing lipin1 (red) and HCV antigen (green) staining. Nuclei were stained using DAPI. Scale bar 10 μm. (B) Representative images showing lack of colocalization of lipin2 signal with that of viral NS proteins. (C) Colocalization analysis of viral antigens with lipin1 and lipin2 showing Pearson´s correlation as well as Mander´s overlap coefficients. Data are shown as average and SD of analysis of 20 regions of interest (ROI) for lipin1 and 10 ROI for lipin2. (TIF) [file ppat.1007284.s001.tif]

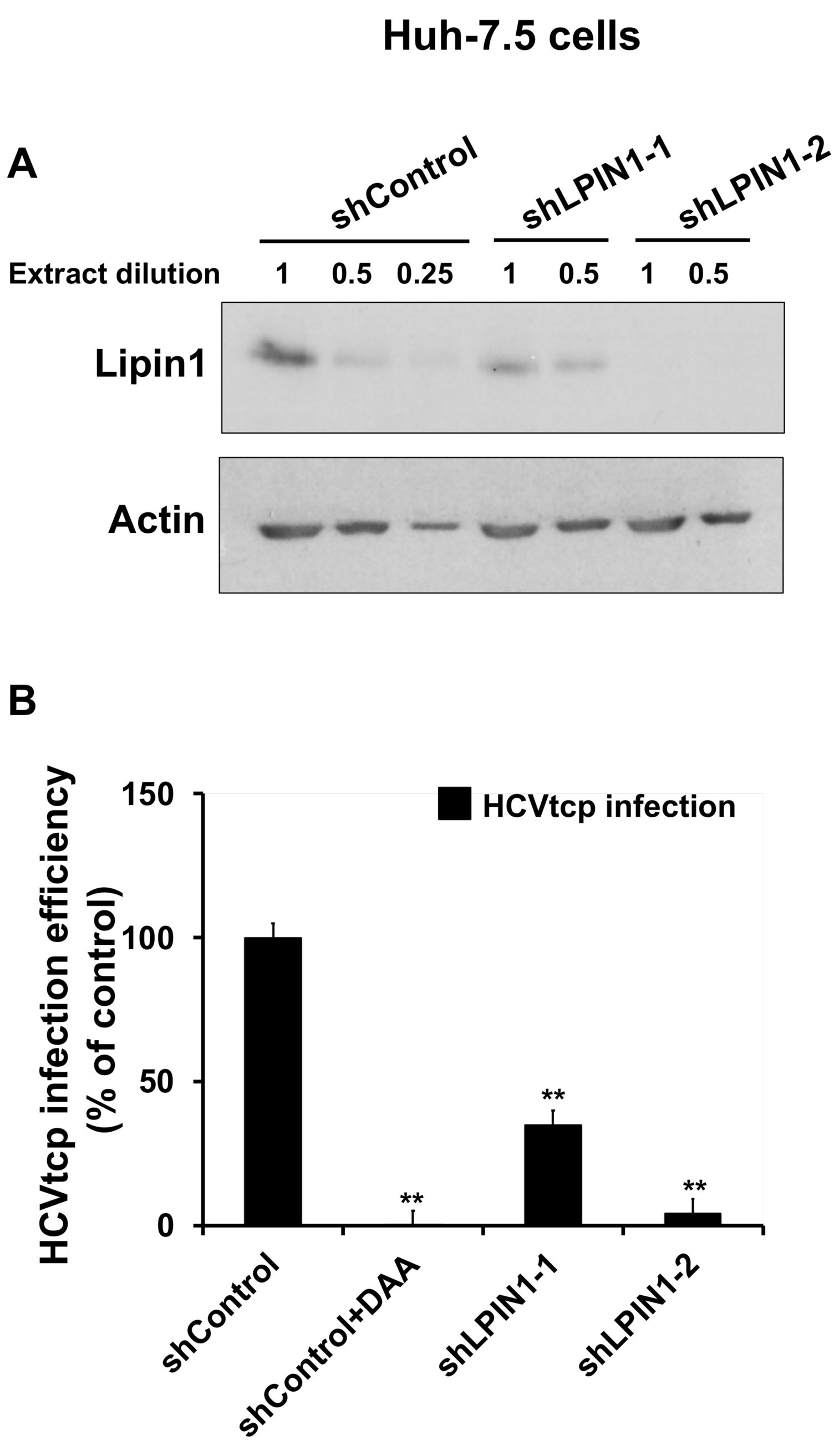

Supplement: S2 Fig — Huh-7.5 cells were transduced with control and lipin1-specific shRNA expressing lentiviral vectors. (A) Total protein samples were collected at day 7 post-transduction, serially diluted and subjected to SDS-PAGE and Western-Blot using antibodies against lipin1 and actin as loading control. (B) Lipin1-deficient Huh-7.5 were subjected to genotype 2a HCVtcp infection. Parallel shControl cell cultures were treated with 10μM 2mAde at the time of infection and cultured in the presence of the inhibitor until the end of the experiment (shControl+DAA). Relative infection efficiency is shown as mean and SD of six experiments performed in triplicate (n = 18). Statistical significance was determined using Student´s t-test (*p<0.05; **p<0.01). (TIF) [file ppat.1007284.s002.tif]

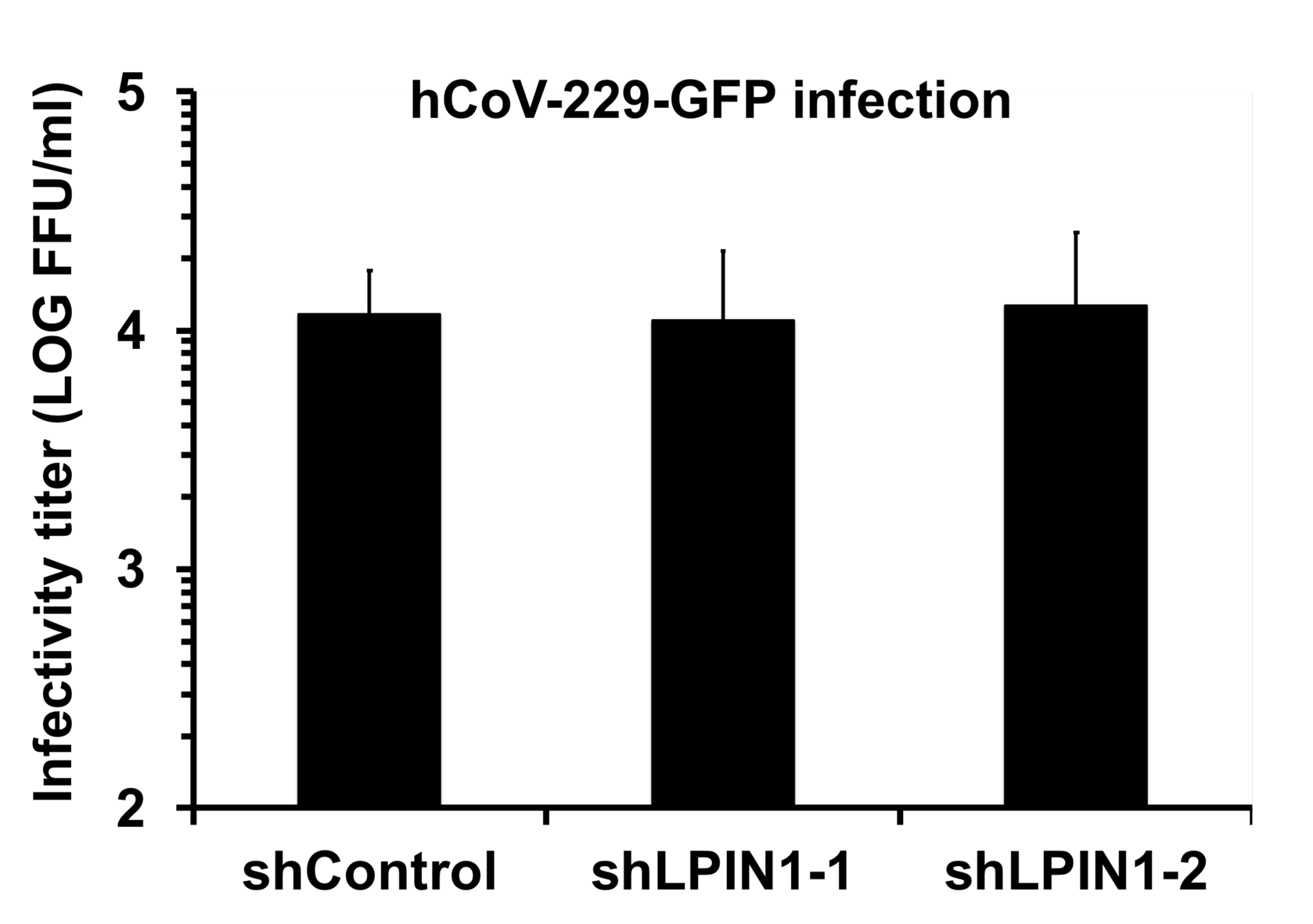

Supplement: S3 Fig — Control and lipin1-deficient Huh-7 cells were inoculated with CoV-229E at MOI 0.01. Supernatants were collected 48 hours post-infection and viral spread was estimated by extracellular infectivity titration. Data are shown as average and SD of three independent experiments performed in triplicate (n = 9). Statistical significance was determined using Student´s t-test (*p<0.05; **p<0.01). (TIF) [file ppat.1007284.s003.tif]

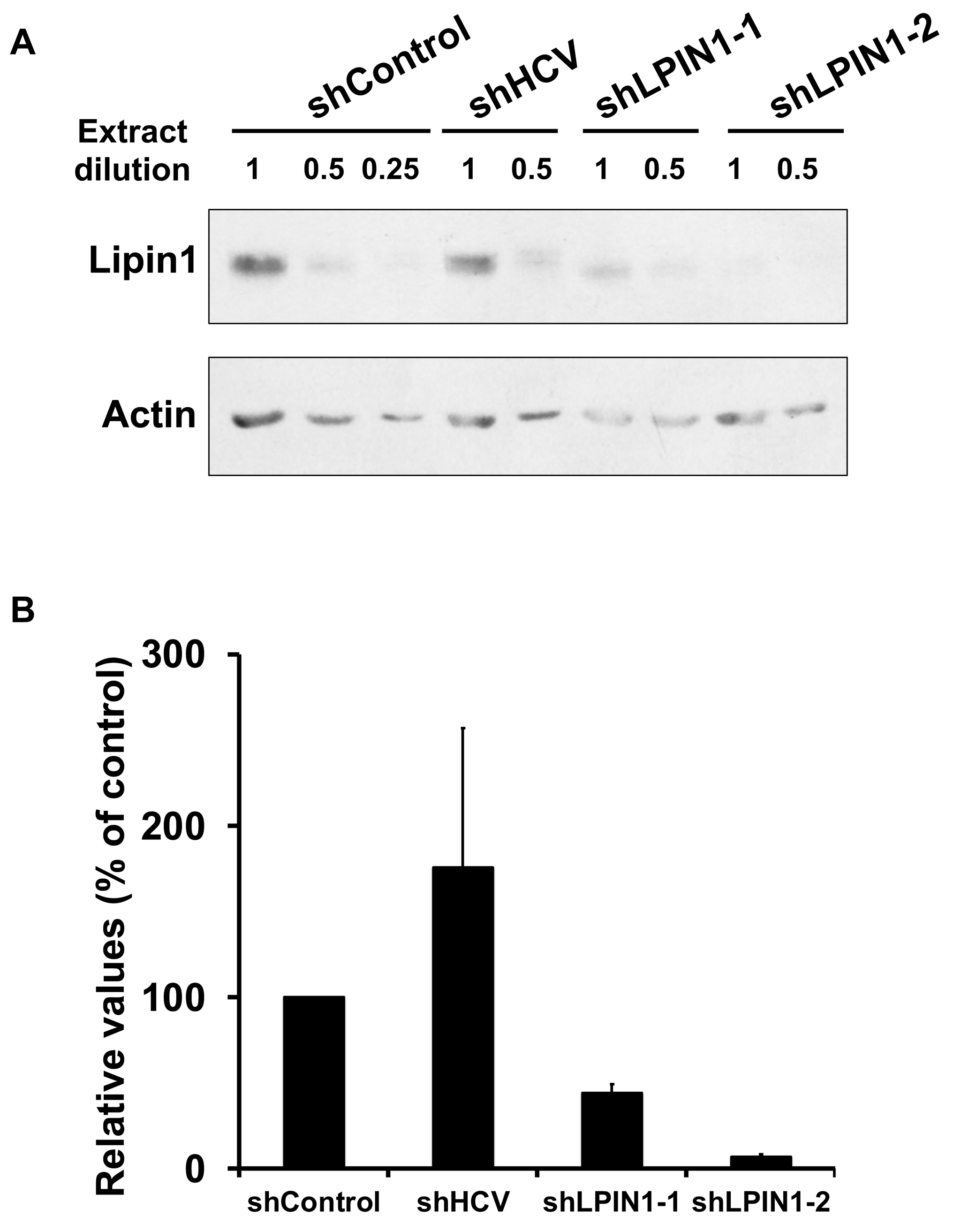

Supplement: S4 Fig — Persistently infected cultures were generated by inoculation with JFH-1 virus at MOI 0.01. Once cultures reached >95% of HCV-positive cells, they were transduced with lentiviral vectors expressing control, HCV RNA-targeting or LPIN1-specific shRNAs. At day 7 post-transduction, cells were harvested to verify lipin1 silencing by Western-Blot using antibodies against lipin1 and actin as loading control. Extracts were serially diluted to facilitate quantitation. (A) Representative Western-Blot. (B) Quantitation of lipin1 levels in the different cell lines. Data are shown as mean and SD two independent experiments (n = 2). (TIF) [file ppat.1007284.s004.tif]

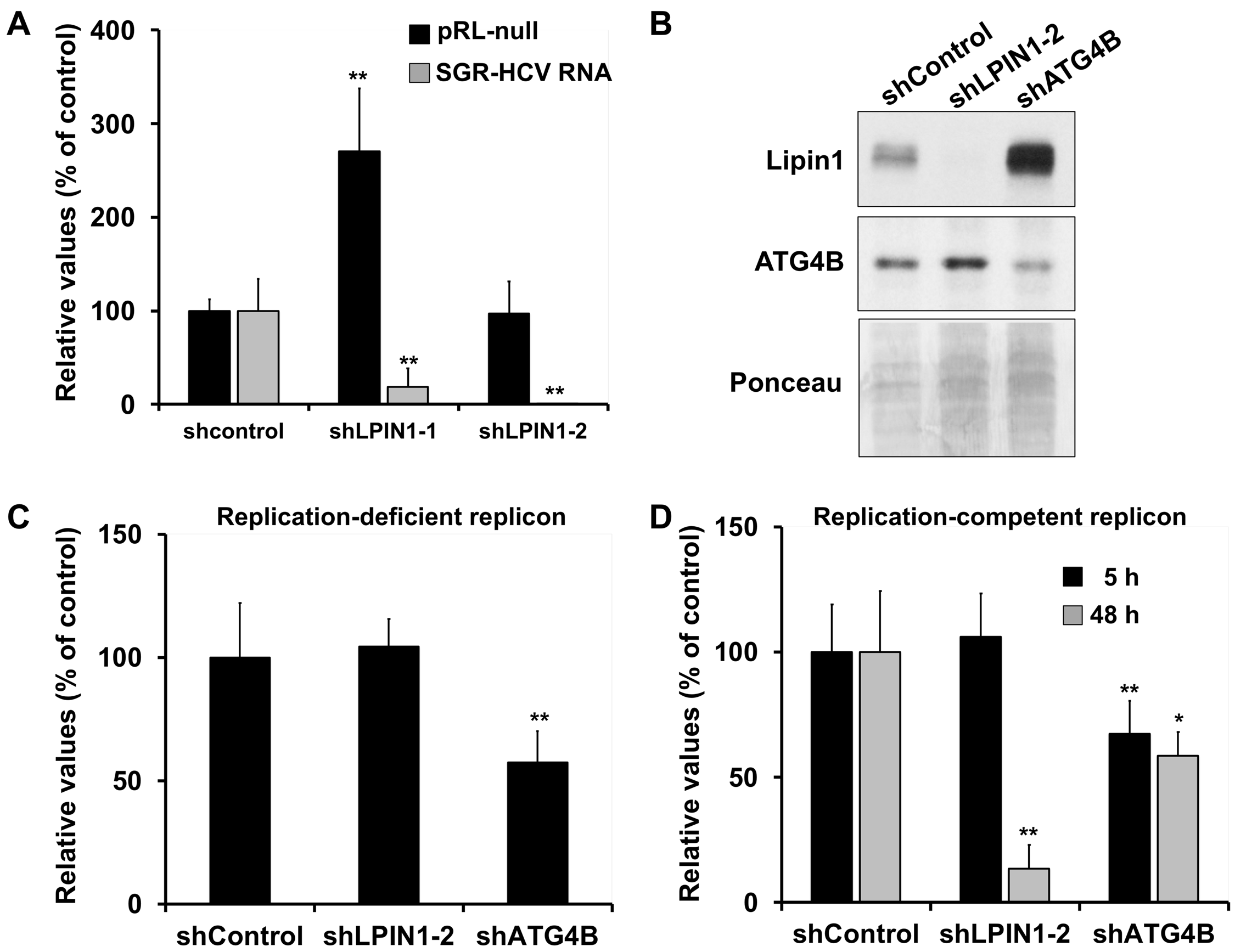

Supplement: S5 Fig — Lipin1-deficient cells were co-transfected with HCV subgenomic replicon bearing Firefly luciferase gene and a plasmid encoding Renilla luciferase. Dual luciferase activity was measured in samples of the transfected cell lines 48 hours post-transfection. (A) Relative plasmid-derived Renilla luciferase as well as SGR replicon-derived Firefly luciferase values are shown as mean and SD of two independent experiments performed in triplicate (n = 6). (B) Lipin1 and ATG4B-deficient cell populations (shLPIN1-2 and shATG4B) were produced by lentiviral transduction. Specific silencing was verified by Western-blot in the different cell lines at day 7 post-transduction. Lipin1 and ATG4B-deficient cells were transfected with a replication-deficient mutant (C) or replication competent subgenomic HCV replicon bearing a luciferase gene (D). Luciferase activity was determined in the different cell lines at 5 hours post-transfection for both replicons and 48 hours post-transfection for the replication-competent replicon RNA. Data are expressed as average and SD of three independent experiments performed in triplicate (n = 9). Statistical significance was determined using Student´s t-test (*p<0.05; **p<0.01). (TIF) [file ppat.1007284.s005.tif]

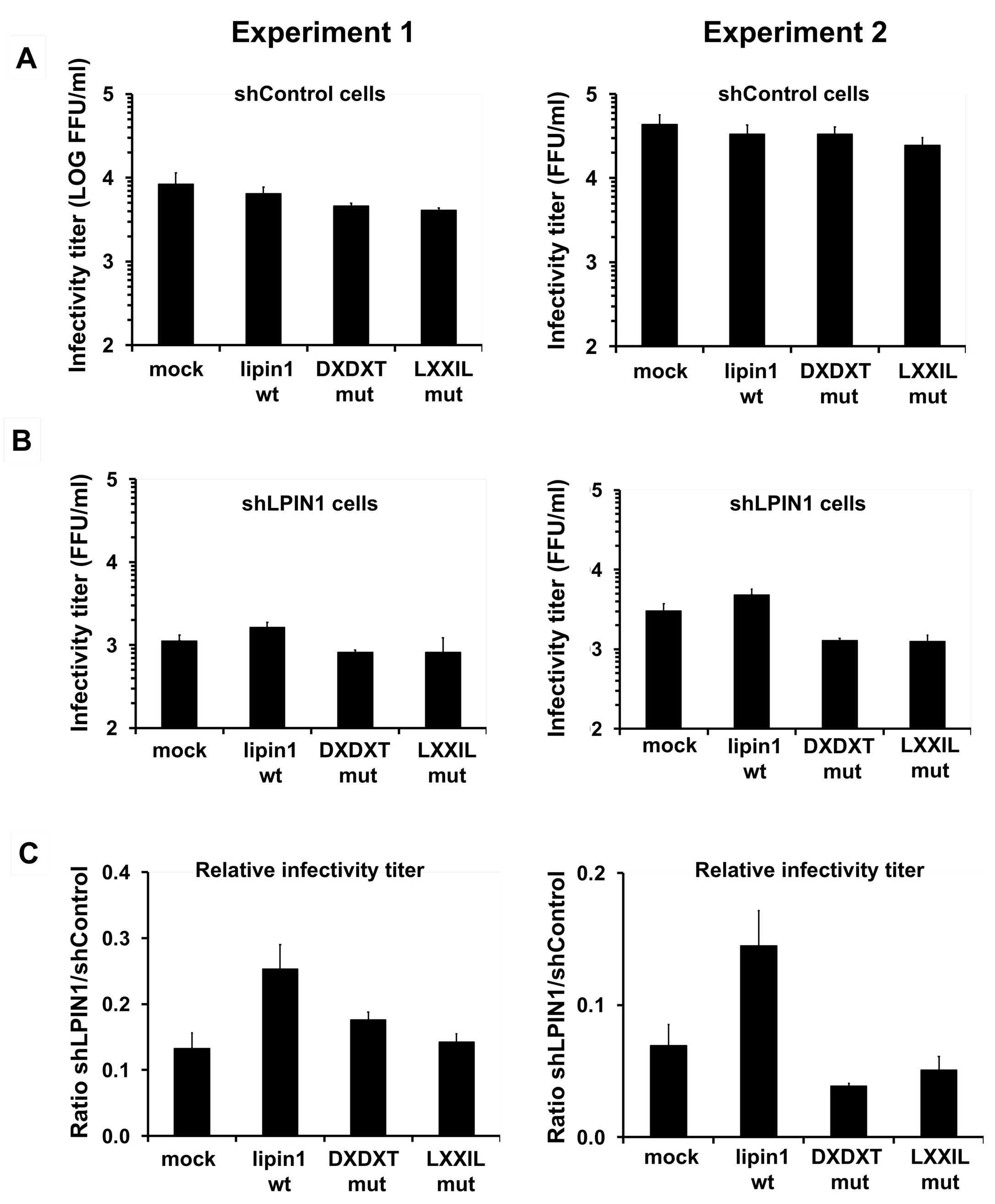

Supplement: S6 Fig — Huh-7 cells were transduced with lentiviral vectors expressing control or LPIN1-specific shRNAs. At day 3 post-transduction, cells were transfected with plasmids expressing wt, DXDXT or LXXIL lipin1beta cDNA. Forty-eight hours later cells were infected at MOI 10 with HCV D183. Two independent experiments are shown (left column; Experiment 1 and right column; Experiment 2). Extracellular infectivity titers were determined in the supernatants 48 hours post-infection. Extracellular infectivity titers determined 48 hours post-infection in shControl (A) and shLPIN1 cells (B). (C) Ratio between the infectivity found in shLPIN1 versus shControl cells in each cell line. (TIF) [file ppat.1007284.s006.tif]

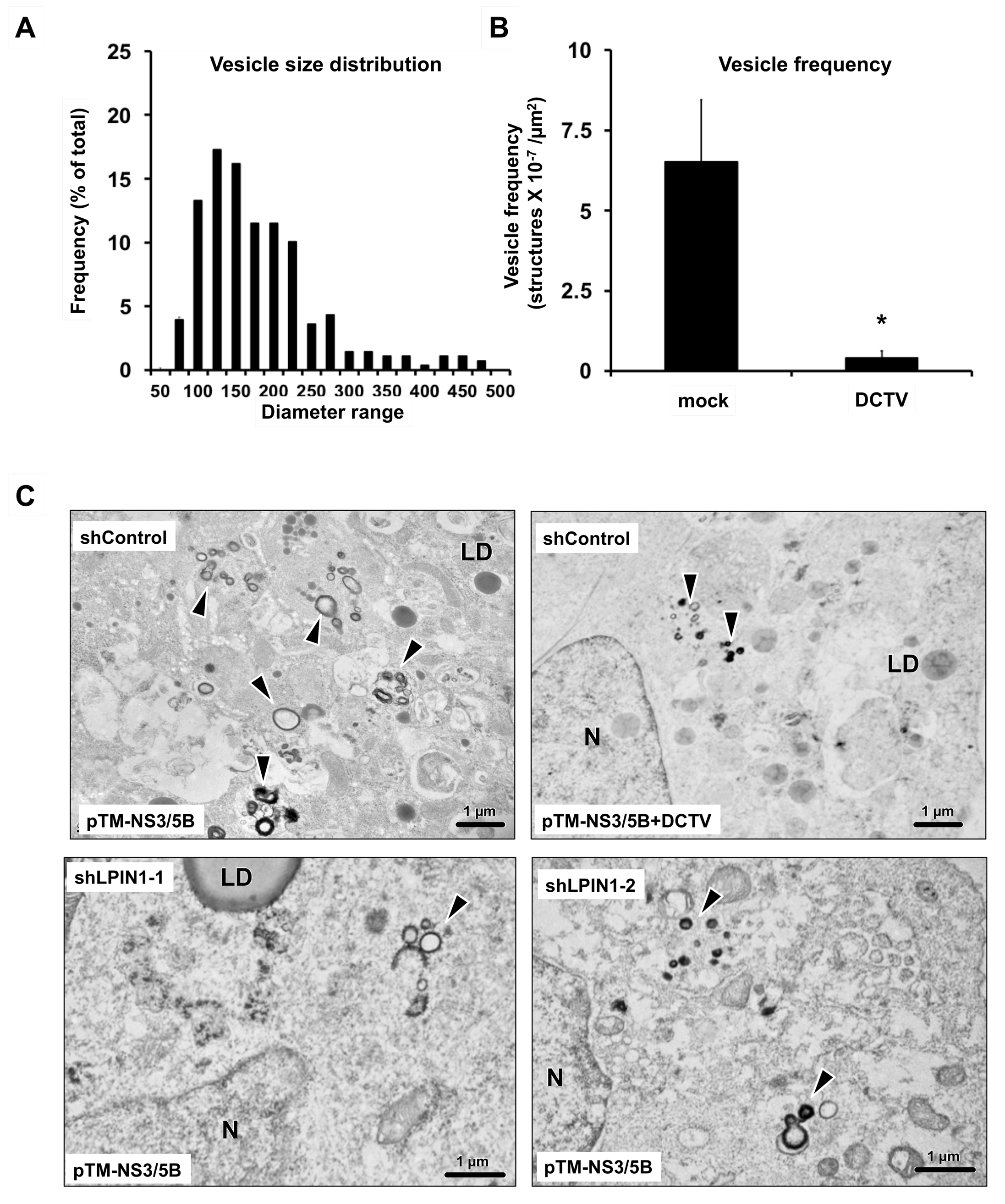

Supplement: S7 Fig — (A) Vesicle size range distribution in shControl mock-treated cells. (B) Frequency of HCV-related structures in mock or DCTV-treated shControl cells expressed as the number of vesicles per cell section surface (μm2). Data are shown as average and SD of 6 (mock-treated) and 10 different cells (DCTV-treated). Statistical significance was determined using Student´s t-test (*p<0.05; **p<0.01). (C) TEM images showing general views of the different cell lines expressing HCV polyprotein (pTM-NS3/5B). DCTV, daclatasvir. (TIF) [file ppat.1007284.s007.tif]

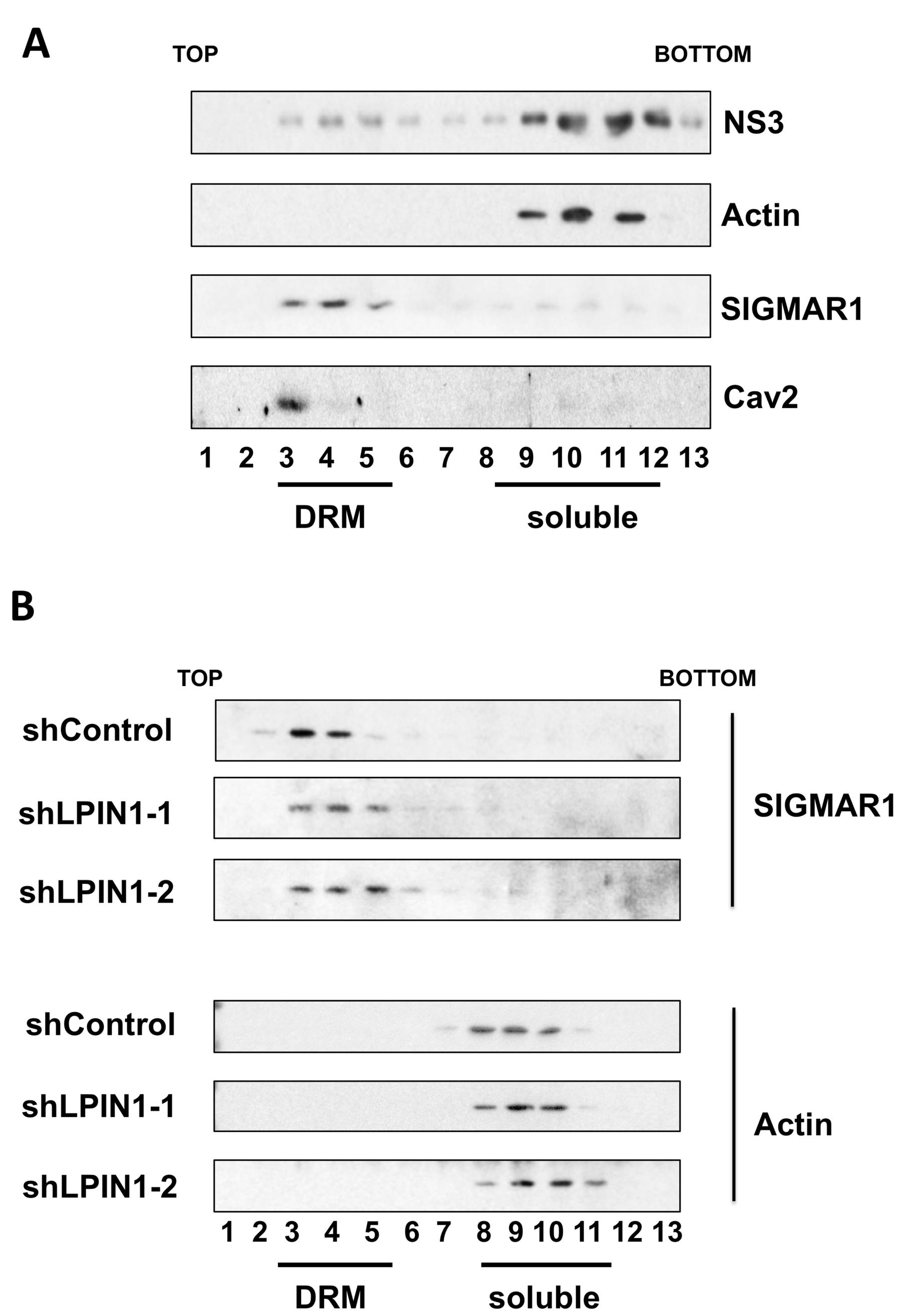

Supplement: S8 Fig — HCV polyprotein was expressed in shControl cells by VacT7 infection and pTM-NS3/5B plasmid transfection. Sixteen hours post-transfection, cells were lysed in TNE-0.1% Triton X114 and clear cell lysates were subjected to isopycnic ultracentrifugation in 10–40% sucrose gradients at 120, 000g during 16 hours at 4°C. (A) Gradient fractions were subjected to SDS-PAGE and Western Blot to determine distribution of NS3, the detergent-soluble protein beta-actin and the DRM markers SIGMAR1 and caveolin-2 (Cav-2). (B) Isopycnic ultracentrifugation of lipin1-deficient cell lysates showing normal sedimentation of DRM (SIGMAR1) and soluble protein markers. (TIF) [file ppat.1007284.s008.tif]
